# Aging Impairs Intramuscular Collagen Remodeling Responses to Repeated Passive Stretching in Skeletal Muscle

**DOI:** 10.3390/ijms27062753

**Published:** 2026-03-18

**Authors:** Yuji Kanazawa, Kenichiro Miyahara, Tatsuo Takahashi, Ryo Miyachi, Mamoru Nagano, Satoshi Koinuma, Naoya Iida, Takao Inoue, Yasufumi Shigeyoshi

**Affiliations:** 1 Department of Physical Therapy, Hokuriku University, Kanazawa 920-1180, Japan; k-miyahara@hokuriku-u.ac.jp (K.M.); ry_miyachi@hokuriku-u.ac.jp (R.M.); 2 Well-Being Research Team, Hokuriku University, Kanazawa 920-1180, Japan; t-takahashi@hokuriku-u.ac.jp; 3 Department of Anatomy and Neurobiology, Faculty of Medicine, Kindai University, Sakai 590-0197, Japan; m-nagano@med.kindai.ac.jp (M.N.); koi@med.kindai.ac.jp (S.K.); shigey@med.kindai.ac.jp (Y.S.); 4 Department of Clinical Pharmacology, Hokuriku University, Kanazawa 920-1181, Japan; 5 Department of Physical Therapy, Fukushima Medical University, Fukushima 960-8516, Japan; iidan@fmu.ac.jp; 6 Department of Pathology, Faculty of Medicine, Kindai University, Sakai 590-0197, Japan; takao@med.kindai.ac.jp

**Keywords:** aging, stretching, extracellular matrix, lysyl oxidase, matrix metalloproteinase

## Abstract

Aging is associated with changes in intramuscular collagen structure and metabolism, which may impair mechanical adaptability and regenerative capacity. We investigated the effects of aging and repeated passive stretching on intramuscular collagen remodeling in the tibialis anterior muscles of mice. The tibialis anterior muscles of young and aged mice were exposed to repeated passive stretching, and the localization of collagen and collagen-related factors was evaluated. Baseline gene expression of collagens I and IV was significantly reduced in aged muscles and was not restored by stretching. Repeated passive stretching increased the area and intensity of collagen I immunoreactivity in both young and aged mice but produced little change in collagen IV. Stretch-induced dynamic changes in lysyl oxidase-positive cells in the extracellular matrix (ECM) were evident in young mice but were markedly attenuated in aged mice. In addition, matrix metalloproteinases (MMP2 and MMP9) mRNA and protein expressions did not differ between groups. No significant age- or stretch-dependent changes were observed in the localization of advanced glycation end products. These findings suggest that although the increase in fibrillar collagen in response to stretching is maintained with aging, the regulatory mechanisms controlling ECM stabilization, particularly those related to cross-linking dynamics, may be impaired.

## 1. Introduction

The extracellular matrix (ECM) of skeletal muscle is a multilayered structure composed of the basement membrane, endomysium, perimysium, and epimysium [1,2]. Collagen I is the most abundant collagen in skeletal muscle and is localized in the endomysium, perimysium, and epimysium [1,3]. Collagen I is fibrous and provides tensile strength and rigidity to muscle fibers [1]. Although collagen I plays a crucial role in maintaining normal skeletal muscle function and structure, excessive accumulation is observed in aging and fibrotic conditions, leading to a decline in muscle quality [2,4]. Furthermore, multiple types of collagens exist within skeletal muscle, including non-fibrous collagens [1]. Collagen IV is localized in the basement membrane and is known as reticular collagen [1,5]. Collagen IV is located closest to muscle fibers and has a protective function [1,5]. Although collagen IV is important for recovery of atrophied muscles, reports indicate that its gene expression is suppressed with aging, potentially delaying muscle recovery [6]. Therefore, collagens I and IV are crucial for maintaining skeletal muscle structure and function.

Collagen remodeling is regulated by the balance between collagen synthesis, collagen cross-linking, and collagen degradation. Collagen cross-linking is divided into enzymatic and non-enzymatic pathways [7]. Enzymes involved in enzymatic cross-linking include lysyl oxidase (LOX) [7]. LOX catalyzes the cross-linking of collagen fibers, thereby enhancing the tensile strength and mechanical stability of the ECM [8,9]. Non-enzymatic collagen cross-linking involves advanced glycation end products (AGEs). With aging, AGEs accumulate in ECM components, including collagen, potentially promoting non-enzymatic collagen cross-linking. This accumulation may contribute to increased stiffness in muscle connective tissue, suggesting it as a factor in age-related decline in muscle function [10,11]. Conversely, collagen degradation is primarily mediated by matrix metalloproteinases (MMPs), particularly MMP-2 and MMP-9, which are essential for ECM metabolic turnover and adaptive remodeling following mechanical stimulation [12,13].

Stretching is a widely used exercise therapy in clinical settings and sports, with practical recommendations summarized in an international expert consensus [14]. However, further research is needed on the adaptive mechanisms of fascia and connective tissues in response to stretching [14]. Thus, stretching represents a clinically relevant yet mechanistically underexplored mechanical stimulus for intramuscular ECM remodeling. Previous studies using experimental animals have reported that passive stretching suppresses the expression of collagen I and IV in skeletal muscle and may contribute to ECM remodeling [15,16]. In aged mice after muscle injury and during aging, imbalances or impairments in ECM remodeling may lead to the accumulation of intramuscular collagen, potentially contributing to a decline in muscle quality [2,4]. Early intervention through exercise before pathology becomes apparent is considered important to prevent muscle aging [17,18,19]. Furthermore, intramuscular collagen accumulates gradually with aging [2], and fibrosis is difficult to address once it has progressed [20], suggesting the importance of preventive intervention. Exercise therapy, a safe nonpharmacological intervention, has garnered attention for preventing skeletal muscle fibrosis [20]. However, the optimal exercise load and exercise modalities remain poorly understood. Therefore, investigating whether stretching—which may promote intramuscular collagen remodeling—can help prevent muscle aging is valuable for understanding muscle aging pathology and developing preventive interventions.

Previous studies examining the effects of repeated passive stretching on intramuscular collagen have used young animals [15,16]. Comparisons between young and aged animals regarding the effects of repeated passive stretching on collagen-related gene expression, collagen localization, cross-linking factor localization, and MMP expression are extremely limited. The temporal dynamics of ECM remodeling in aged skeletal muscle in response to repeated passive stretching remain largely unexplored. Therefore, we aimed to investigate the short-term effects of aging and repeated passive stretching on intramuscular collagen remodeling in the tibialis anterior muscle of young and aged mice.

## 2. Results

### 2.1. Body Weight, Muscle Weight, Relative Muscle Weight, and Fiber Cross-Sectional Area

**Figure 1:**
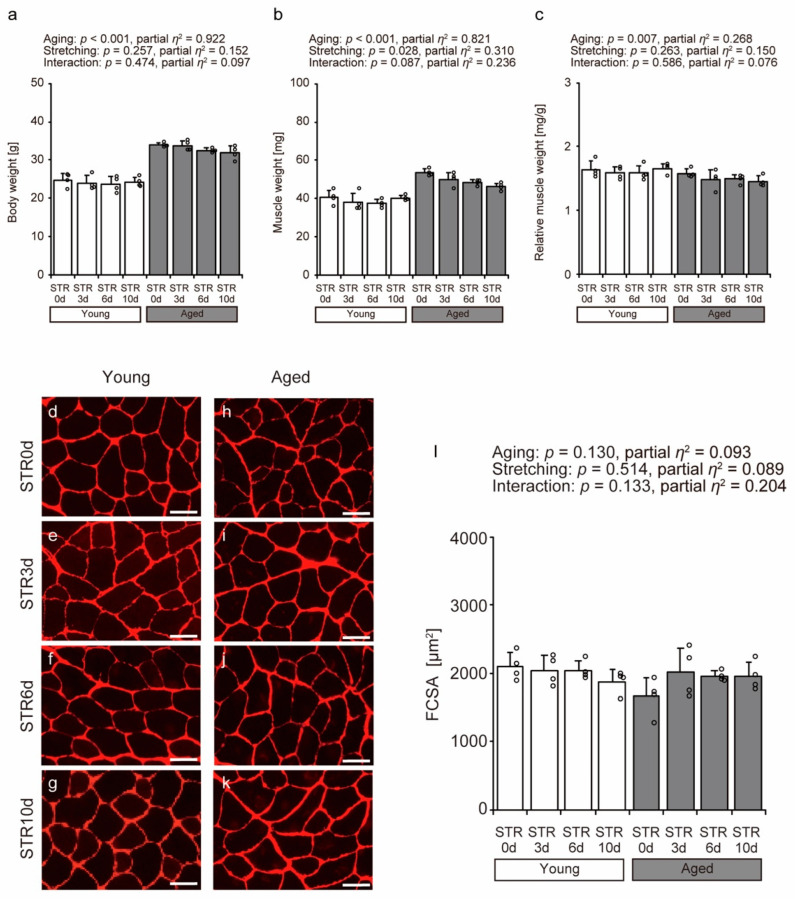
Effects of aging and stretching on body weight (**a**), the tibialis anterior muscle weight (**b**), relative muscle weight normalized by body weight (**c**), and fiber cross-sectional area (FCSA; (**d**–**l**)) in young and aged mice. Transverse sections of the tibialis anterior muscle were prepared and visualized using immunofluorescence with anti-collagen VI antibody to outline muscle fibers (**d**–**k**). The scale bar indicates 50 µm. FCSA was measured using the stained images (**l**). Bar dot plots were created using means, with error bars indicating standard deviation and individual data points displayed as dots on histograms for each group (*n* = 4 per group). White bars represent young mice, and gray bars represent aged mice. Two-way ANOVA was performed. No significant interaction between age and stretching was observed; therefore, post hoc multiple comparisons were not conducted, with *p* < 0.05 considered significant (**a**–**c**,**l**). The graph shows the *p*-value and effect size (partial *η*^2^). STR0d: No stretching, STR3d: Stretching for 3 days, STR6d: Stretching for 6 days, STR10d: Stretching for 10 days.

Bodyweight, muscle weight, and relative muscle weight were examined to investigate the effects of aging and stretching on the tibialis anterior muscle (Figure 1a–c). With aging, body weight and muscle weight increased (Figure 1a,b), whereas relative muscle weight decreased (Figure 1c). Furthermore, stretching had minimal effects on body weight, muscle weight, and relative muscle weight. In addition, to investigate the effects of aging and stretching on muscle fibers, we measured the fiber cross-sectional area (FCSA) of the tibialis anterior muscle using immunofluorescence (IF) images stained with anti-collagen VI antibodies (Figure 1d–l). The results showed that aging and stretching had no significant effect on FCSA (Figure 1l). These findings indicate that the aged mice used in this study were in a pre-atrophy stage, before the onset of significant age-related muscle atrophy. Furthermore, the stretching protocol employed in this study did not significantly affect muscle fiber size.

### 2.2. Collagen-Related Factors

**Figure 2:**
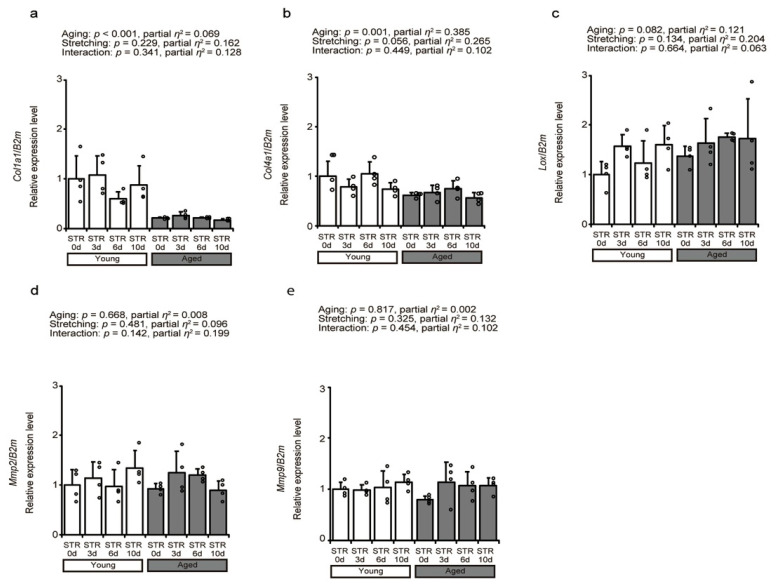
Effects of aging and stretching on collagen-related factor expression in young and aged mice. Relative mRNA expression levels of *Col1a1* (**a**), *Col4a1* (**b**), *Lox* (**c**), *Mmp2* (**d**), and *Mmp9* (**e**) were measured using quantitative PCR. Expression levels were normalized against the housekeeping gene, beta-2 microglobulin (*B2m*). Bar dot plots were created using mean values; error bars indicate standard deviation, and individual data points are displayed as dots on the histogram for each group (*n* = 4 per group). White bars represent young mice, and gray bars represent aged mice. Two-way ANOVA was performed. No significant interaction between age and stretching was observed; therefore, post hoc multiple comparisons were not conducted, with *p* < 0.05 considered significant (**a**–**e**). The graph shows the *p*-value and effect size (partial *η*^2^). STR0d: No stretching, STR3d: Stretching for 3 days, STR6d: Stretching for 6 days, STR10d: Stretching for 10 days.

The effects of aging and stretching on the expression of intramuscular collagen genes were investigated by measuring changes in collagen-related factor expression using quantitative PCR (Figure 2a–e). The mRNA expression levels of *Col1a1* and *Col4a1,* which encode collagen I and IV, respectively, decreased with aging; however, no significant changes were observed with stretching (Figure 2a,b). Next, *Lox* expression, which contributes to collagen cross-linking and muscle regeneration, showed no significant effects of aging or stretching (Figure 2c). Finally, the expression of MMP2 and MMP9, which are known collagen-degrading enzyme genes, was examined (Figure 2d,e). No significant effects of aging and stretching on *Mmp2* and *Mmp9* expression were observed (Figure 2d,e).

### 2.3. Collagen I Localization

**Figure 3:**
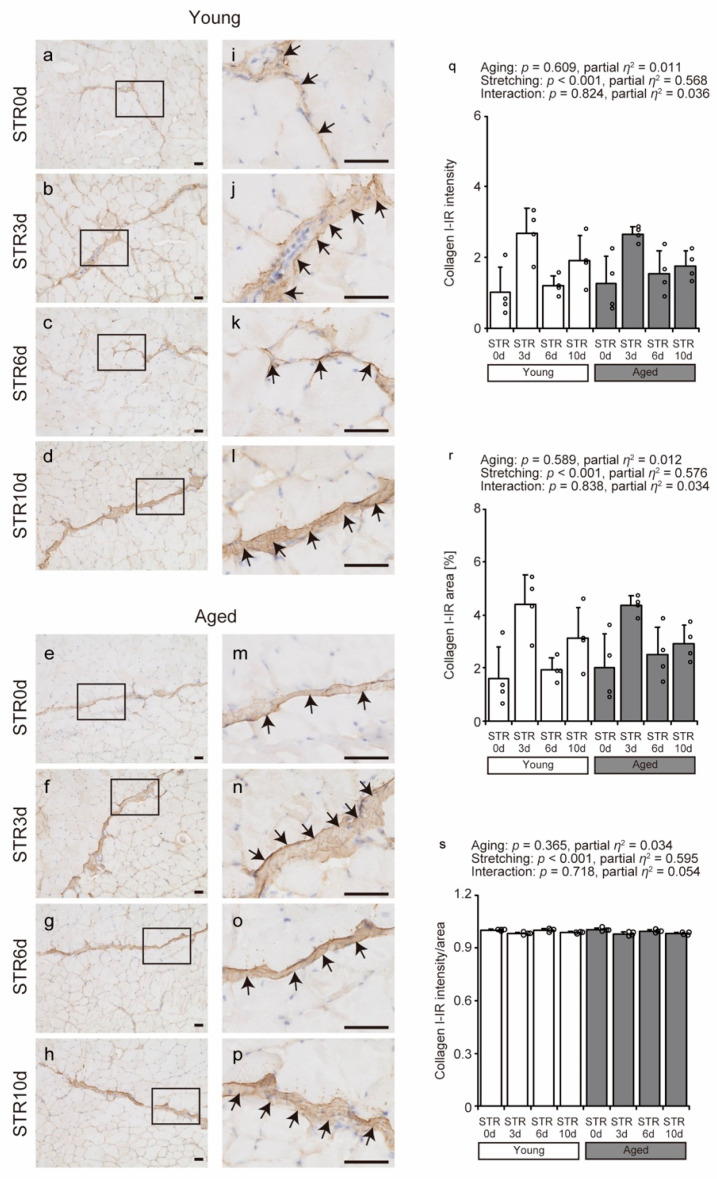
Effects of aging and stretching on collagen I localization in young and aged mice. Collagen I was visualized using immunohistochemical staining with anti-collagen I antibody in transverse sections of the tibialis anterior muscle, with hematoxylin used for counterstaining (**a**–**p**). Magnified views of rectangular regions in (**a**–**h**) are shown for detailed observation (**i**–**p**). Scale bar = 50 μm. Collagen I immunoreactivity (IR) was primarily detected in the perimysium across all groups (arrows in (**i**–**p**)). Collagen I IR intensity, area, and intensity/area were quantified using stained images (**q**–**s**). Bar dot plots were created using means; error bars indicate standard deviation, and individual data points are shown as dots on histograms for each group (*n* = 4 per group). White bars represent young mice, and gray bars represent aged mice. Two-way ANOVA was performed. No significant interaction between age and stretching was observed; therefore, post hoc multiple comparisons were not conducted, with *p* < 0.05 considered significant (**q**–**s**). The graph shows the *p*-value and effect size (partial *η*^2^). STR0d: No stretching, STR3d: Stretching for 3 days, STR6d: Stretching for 6 days, STR10d: Stretching for 10 days.

Immunohistochemistry (IHC) was used to visualize the localization of collagen I, the most abundant and representative fibrillar collagen in intramuscular tissue. In all groups, collagen I was primarily localized in the perimysium and partially localized in the endomysium (Figure 3a–p). The area and intensity of collagen I immunoreactivity (IR) did not change with aging but increased with stretching (Figure 3q,r). However, when normalized to the stained area, the IR intensity per unit area showed a small but statistically significant decrease with stretching (Figure 3s). These findings indicate that the stretching-induced increase in collagen I IR mainly reflects an expansion of the collagen I–positive area rather than an increase in signal intensity per unit area in either young or aged mice.

### 2.4. Collagen IV Localization

**Figure 4:**
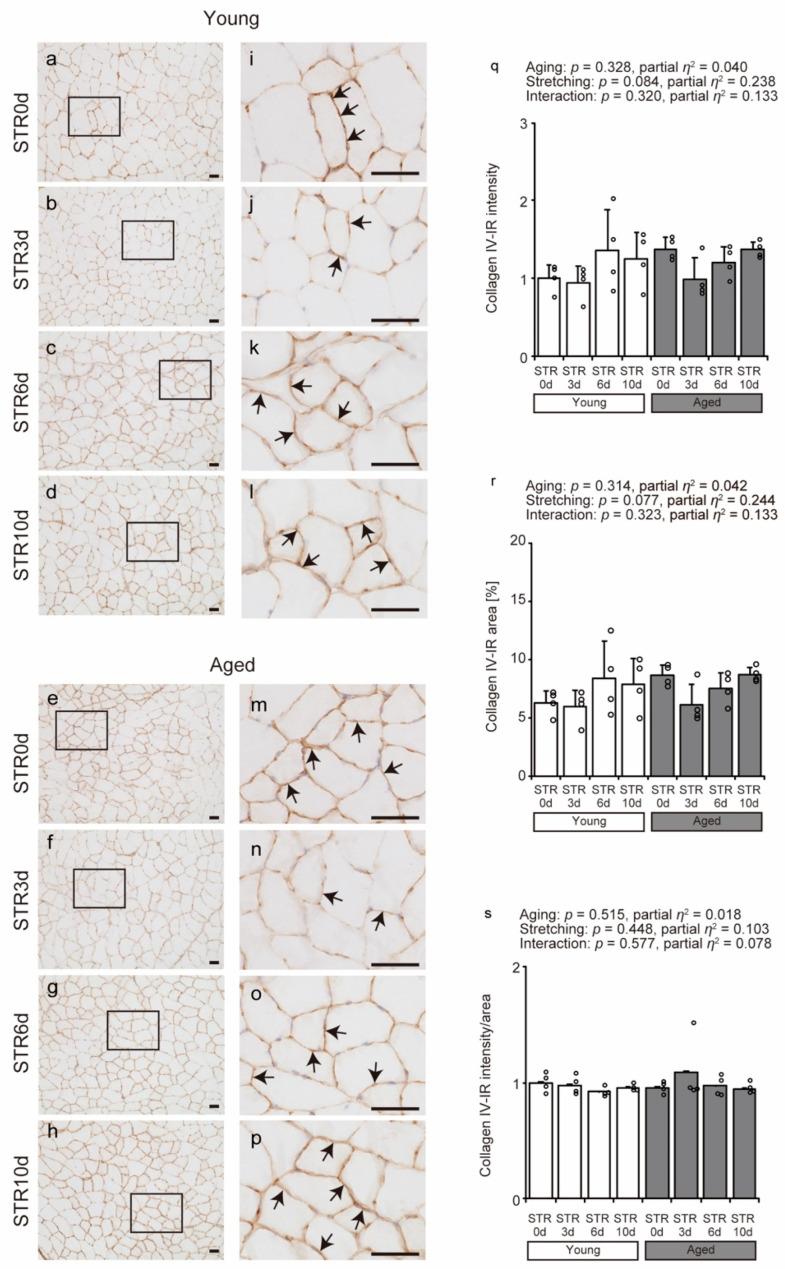
Effects of aging and stretching on collagen IV localization in young and aged mice. Collagen IV was visualized using immunohistochemical staining with anti-collagen IV antibody in transverse sections of the tibialis anterior muscle, with hematoxylin used for counterstaining (**a**–**p**). Magnified views of rectangular regions in (**a**–**h**) are shown for detailed observation (**i**–**p**). Scale bar = 50 μm. Collagen IV immunoreactivity (IR) was primarily detected in the basement membrane across all groups (arrows in (**i**–**p**)). Collagen IV IR intensity, area, and intensity/area were quantified using stained images (**q**–**s**). Bar dot plots were created using means; error bars indicate standard deviation, and individual data points are shown as dots on histograms for each group (*n* = 4 per group). White bars represent young mice, and gray bars represent aged mice. Two-way ANOVA was performed. No significant interaction between age and stretching was observed; therefore, post hoc multiple comparisons were not conducted, with *p* < 0.05 considered significant (**q**–**s**). The graph shows the *p*-value and effect size (partial *η*^2^). STR0d: No stretching, STR3d: Stretching for 3 days, STR6d: Stretching for 6 days, STR10d: Stretching for 10 days.

IHC was used to visualize the localization of collagen IV, a reticular collagen found in the basement membrane. In all groups, collagen IV was primarily localized to the basement membrane (Figure 4a–p). Collagen IV IR intensity, area, and intensity/area showed no change with aging and stretching (Figure 4q–s). These results suggest that collagen IV localization and IR were not affected by either aging or stretching under the present experimental conditions in young and aged individuals.

### 2.5. AGE Localization

**Figure 5:**
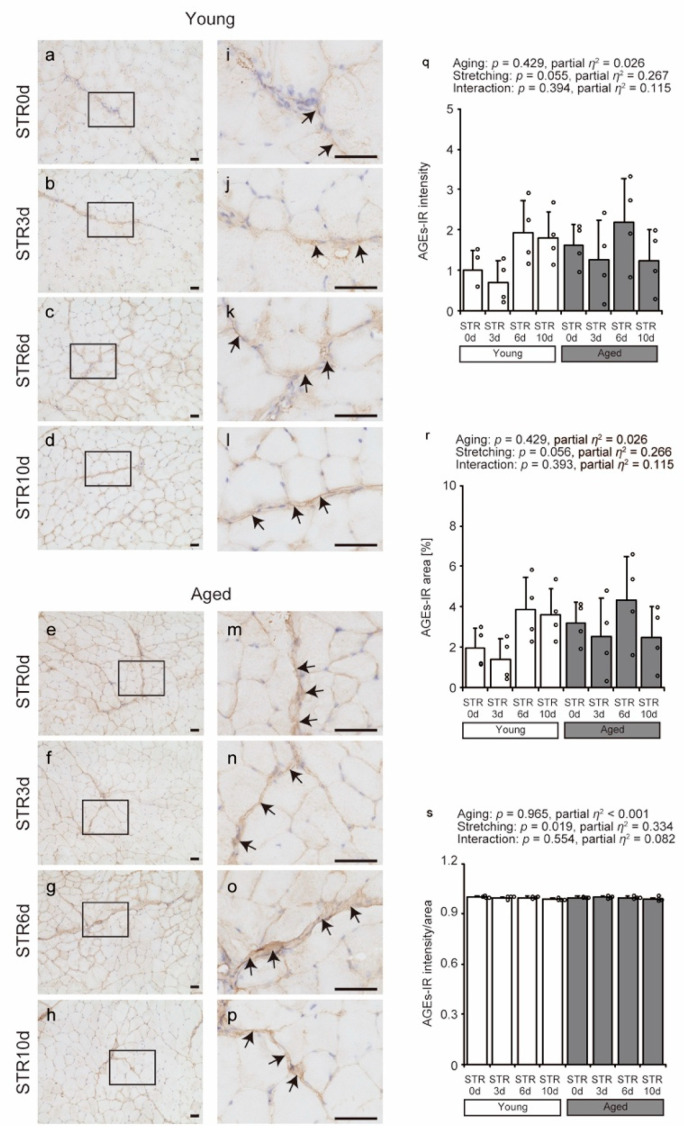
Effects of aging and stretching on AGE localization in young and aged mice. AGEs were visualized using immunohistochemical staining with anti-AGE antibody in transverse sections of the tibialis anterior muscle, with hematoxylin used for counterstaining (**a**–**p**). Magnified views of rectangular regions in (**a**–**h**) are shown for detailed observation (**i**–**p**). Scale bar = 50 μm. AGE immunoreactivity (IR) was primarily detected in the perimysium across all groups (arrows in (**i**–**p**)). AGE IR intensity, area, and intensity/area were quantified using stained images (**q**–**s**). Bar dot plots were created using means; error bars indicate standard deviation, and individual data points are shown as dots on histograms for each group (*n* = 4 per group). White bars represent young mice, and gray bars represent aged mice. Two-way ANOVA was performed. No significant interaction between age and stretching was observed; therefore, post hoc multiple comparisons were not conducted, with *p* < 0.05 considered significant (**q**–**s**). The graph shows the *p*-value and effect size (partial *η*^2^). STR0d: No stretching, STR3d: Stretching for 3 days, STR6d: Stretching for 6 days, STR10d: Stretching for 10 days.

IHC was used to visualize the localization of AGEs involved in collagen cross-linking. AGEs were confirmed to be primarily localized in the perimysium in all groups (Figure 5a–p). The area and intensity of AGE IR were not affected by aging or stretching (Figure 5q,r). In contrast, when normalized to the stained area, the IR intensity per unit area showed a significant main effect of stretching (Figure 5s), with no significant effect of age or interaction. These findings indicate a slight reduction in AGE IR density following stretching, without changes in total stained area or overall IR intensity. Although statistically significant, the magnitude of change was small; however, even subtle alterations in ECM-associated AGE density may influence matrix stiffness and muscle quality.

### 2.6. LOX Localization

**Figure 6:**
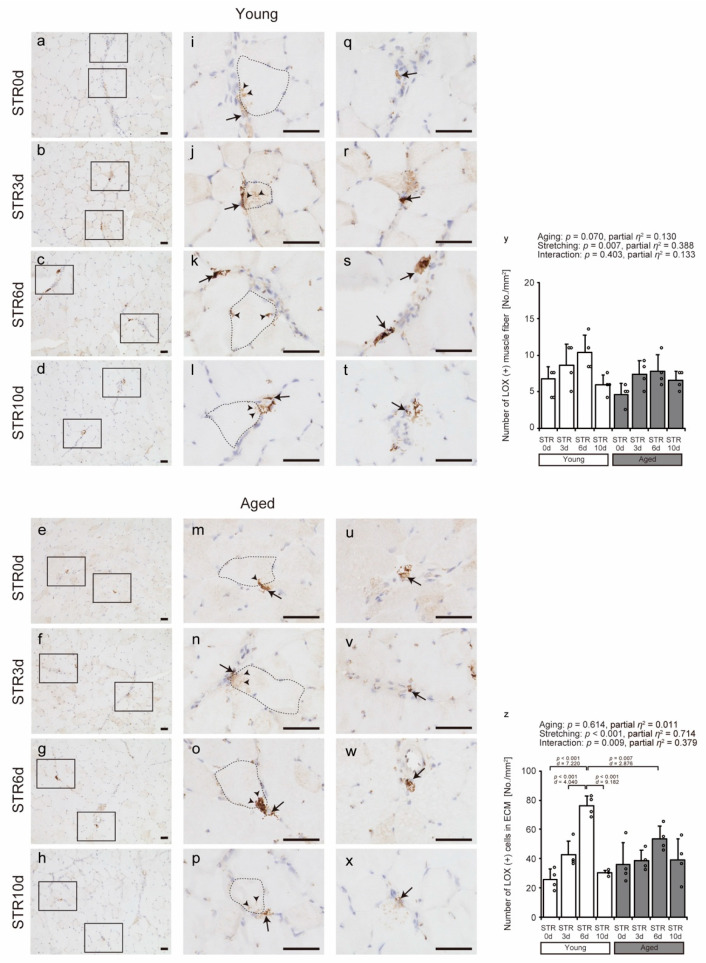
Effects of aging and stretching on the localization of lysyl oxidase (LOX) in young and aged mice. LOX was visualized using immunohistochemical staining with anti-LOX antibody in transverse sections of the tibialis anterior muscle, with counterstaining using hematoxylin (**a**–**x**). Magnified views of rectangular regions in (**a**–**h**) are shown for detailed observation (**i**–**x**). Scale bar = 50 μm. Representative localization of LOX-positive muscle fibers is shown in (**i**–**p**), where muscle fibers are outlined with black dashed lines and cytoplasmic staining is indicated by arrowheads. LOX-positive cells in the ECM are indicated by arrows in (**i**–**x**). The number of LOX-positive muscle fibers per unit area and the number of LOX-positive cells in the ECM were counted (**y**,**z**). Bar dot plots were created using mean values; error bars indicate standard deviation, and individual data points are shown as dots on the histogram for each group (*n* = 4 per group). White bars represent young mice, and gray bars represent aged mice. Statistical analysis was performed using two-way ANOVA with age and stretching as factors. No significant interaction between age and stretching was observed; therefore, post hoc multiple comparisons were not conducted (**y**). A significant interaction between age and stretching was observed; therefore, Tukey’s post hoc multiple comparisons were performed (**z**), with *p* < 0.05 considered significant (**y**,**z**). The graph shows the *p*-value and effect size (partial *η*^2^ or Cohen’s *d*). STR0d: No stretching, STR3d: Stretching for 3 days, STR6d: Stretching for 6 days, STR10d: Stretching for 10 days.

IHC was used to visualize the localization of LOX, which is involved in collagen cross-linking (Figure 6a–x). LOX was localized to the cytoplasm of muscle fibers (arrowheads in Figure 6i–p) and was also identified as LOX-positive cells in the ECM region in all groups (arrows in Figure 6i–x). LOX-positive muscle fibers and cells in the ECM were counted separately (Figure 6y,z). The number of LOX-positive muscle fibers was significantly affected by stretching in both young and aged mice (Figure 6y). Furthermore, a significant age × stretching interaction was detected for the number of LOX-positive cells in the ECM. Tukey’s honest significant difference test revealed that, in young mice, the number of LOX-positive cells in ECM significantly increased at STR3d and STR6d compared with STR0d. The value at STR6d was significantly higher than that at STR10d, indicating a peak at STR6d. In addition, the value in young mice at STR6d was significantly higher than that in aged mice (Figure 6z).

### 2.7. MMP Expression

**Figure 7:**
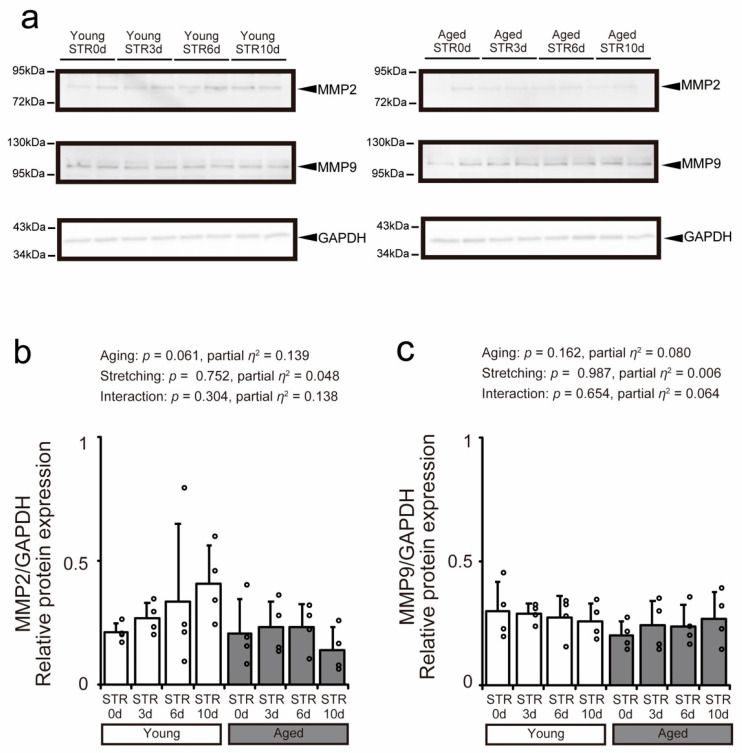
Effects of aging and stretching on matrix metalloproteinase (MMP) expression in young and aged mice. Using the tibialis anterior muscle, MMP2 and MMP9 expression levels, along with glyceraldehyde-3-phosphate dehydrogenase (GAPDH) as an endogenous control, were measured using Western blotting (**a**). The relative expression graph shows bar dot plots using mean values, with error bars indicating standard deviation. Individual data points are displayed as dots on the histogram for each group (*n* = 4 per group). White bars represent young mice, and gray bars represent aged mice. A two-way ANOVA was performed to test for a statistical interaction between age and stretching, with *p* < 0.05 considered significant (**b**,**c**). The graph shows the *p*-value and effect size (partial *η*^2^). STR0d: No stretching, STR3d: Stretching for 3 days, STR6d: Stretching for 6 days, STR10d: Stretching for 10 days.

Western blotting was performed to examine MMP expression in the tibialis anterior muscles of young and aged mice (Figure 7a). No significant changes in MMP2 or MMP9 expression were observed with aging or stretching (Figure 7b,c).

## 3. Discussion

We investigated the effects of aging and repeated passive stretching on intramuscular collagen remodeling in the tibialis anterior muscles of mice. The key findings were as follows: (1) baseline expression of collagen genes was reduced with aging and was not restored by stretching; (2) stretching increased the area and intensity of collagen I IR in both young and aged muscles, whereas no changes were observed for collagen IV; (3) stretching and aging did not significantly affect MMP mRNA or protein expression levels; and (4) the increase in LOX-positive cell numbers in the ECM due to stretching was pronounced in young mice but was markedly attenuated in aged mice. Collectively, these findings suggest that although the response of fibrous collagen to repeated passive stretching is maintained with aging, the regulatory mechanisms controlling ECM stabilization and construction may be impaired.

In this study, collagen I and IV gene expression was significantly reduced in aged muscle. This finding is consistent with previous reports indicating age-related declines in ECM gene transcription and metabolic turnover in skeletal muscles [21,22,23]. Notably, repeated passive stretching failed to restore collagen gene expression in aged mice in this study, suggesting a potential resistance to mechanical stimulus transduction with aging. Aged skeletal muscle has been shown to exhibit blunted transcriptional and signaling responses to mechanical stimuli [24,25]. These blunt transcriptional and signaling responses could limit ECM regeneration and adaptive remodeling, even in the presence of mechanical loading. However, as the stretching stimulus used in this study did not alter collagen gene induction in young muscles, definitively determining whether age-related blunting of the response to mechanical stimuli exists is not possible.

The collagen I IR area and intensity increased with repeated passive stretching regardless of age, whereas no significant changes were observed in the collagen IV IR area and intensity in this study. The difference between the two types of collagens may be attributed to their distinct localization and mechanical roles. Collagen I, a major fibrillar collagen of the ECM that is primarily located in the epimysium and perimysium, plays a central role in force transmission [1,3]. In contrast, collagen IV is localized in the basement membrane region and provides structural stability to muscle fibers [5]. The mechanical stimulus from stretching used in this study likely affected collagen I levels in skeletal muscle. In contrast to this study, previous studies have reported that passive stretching of the soleus muscle reduced the stained area of both collagen I and IV [15,16]. These results suggest that the adaptation to stretching may differ depending on the muscle fiber type. Slow-twitch muscles, such as antigravity muscles, are exposed to physiologically sustained mechanical loading and contain more collagen than that in fast-twitch muscles [24]. As collagen contributes to static tension within skeletal muscle, the expansion of collagen areas is thought to reflect adaptation to sustained mechanical loading. Therefore, the collagen I IR area and intensity observed in the tibialis anterior muscle (fast-twitch dominant) in this study likely reflect an adaptive response of the ECM to repetitive passive stretching. In skeletal muscles, pathological fibrosis is typically associated with expansion of connective tissue in the endomysium and perimysium, muscle fiber atrophy, and dysregulated matrix turnover reflected by altered MMP expression [3]. In the present study, although collagen I IR area and intensity increased following repetitive passive stretching, we did not observe muscle fiber atrophy or significant alterations in MMP expression. These findings indicate that matrix turnover was not markedly dysregulated, and they suggest that the observed ECM changes may represent adaptive remodeling rather than clear evidence of progressive fibrosis. However, because we did not evaluate the persistence of collagen accumulation, biomechanical properties, or collagen cross-link maturity, we cannot conclusively distinguish adaptive ECM reinforcement from early-stage fibrotic remodeling. Future studies incorporating longitudinal assessment and functional analyses will be required to clarify this distinction. In addition, the dissociation between reduced collagen gene expression and increased collagen I IR intensity and area suggests that collagen I expansion may not be driven by enhanced transcription. Instead, post-transcriptional regulation, reduced collagen degradation, or increased stabilization of pre-existing ECM components could underlie the observed protein-level changes. Therefore, collagen I expansion in this context likely reflects altered ECM turnover dynamics rather than increased synthesis. This interpretation is supported by the reduced integrated intensity per unit area, indicating a broader but less dense distribution of collagen I deposition, consistent with spatial redistribution rather than with focal accumulation.

Repeated passive stretching did not significantly affect MMP mRNA or protein expression levels in this study. MMPs play crucial roles in collagen degradation following mechanical or injury-related stimuli, ECM metabolic turnover, and muscle regeneration [26]. Stretching increases MMP expression and promotes intramuscular collagen remodeling [16]. Conversely, the lack of change in MMP expression levels in this study may indicate that the experimental conditions did not promote intramuscular collagen degradation. Intramuscular collagen is maintained by a balance among collagen production, maturation through cross-linking, and degradation [27,28]. This study also revealed that stretching increased the number of LOX-positive cells in the ECM. Collectively, these findings suggest that the passive stretching used in this study may have predominantly induced phases related to collagen maturation—that is, ECM stabilization and construction—rather than promoting MMP-mediated collagen degradation. To address concerns regarding antibody specificity in the Western blot analysis, additional negative control experiments using rabbit IgG were performed (Supplementary Figure S15). Several minor bands were detected in the IgG-probed membranes, indicating non-specific background signals. Notably, the band at the predicted molecular weight was absent in the IgG condition, supporting the specificity of the signal quantified in this study. However, because MMP expression levels were relatively low under the present experimental conditions, the specific signal appeared weak and background bands were relatively more noticeable. Therefore, the Western blot findings should be interpreted with caution.

Similarly, LOX localization analysis revealed that repeated passive stretching increased the number of LOX-positive cells in the ECM of young mice, peaking on day 6. The peak value in aged mice also occurred on day 6 but was significantly lower than that in young mice. LOX-mediated enzymatic cross-linking is essential for collagen maturation and mechanical stability of the ECM [7,8,9]. The blunted LOX response in aged muscles suggests reduced adaptability of collagen cross-linking processes in response to mechanical stimuli. In contrast, no marked changes in AGE localization were detected with aging or stretching in this study. Although AGE accumulation is a characteristic feature of advanced aging [10,11], the aged mice used in this study likely represent a prefibrotic stage before substantial AGE deposition. This interpretation—that the aged mice were in a prefibrotic, pre-atrophic stage—is consistent with results showing relatively preserved muscle weight and minimal age-related muscle atrophy.

Some limitations should be acknowledged. First, only male mice were used in this study. Given that estrogen and other sex hormones influence ECM remodeling and collagen metabolism, sex-dependent differences may exist. Future studies including female mice are warranted. Second, direct torque measurements were not performed, and stretching was standardized based on joint angle rather than on applied force. This approach was adopted to ensure consistent joint positioning across animals and to reflect clinical practice, where passive stretching is typically prescribed according to joint position rather than direct force measurement. However, because aged muscle exhibits altered passive stiffness, the actual mechanical load imposed on the ECM may have differed between age groups despite identical joint angles. Such differences in mechanical strain could influence LOX activation and collagen remodeling responses. Future studies incorporating direct force or torque measurements would further refine the mechanobiological interpretation of stretching-induced ECM adaptations. Third, we focused on short-term stretching, and long-term adaptation remains unknown. Fourth, the underlying mechanism for the discrepancy between collagen I mRNA expression and IR area and intensity was not examined directly. Although stretching may affect the intramuscular collagen structure through the stabilization of the existing ECM rather than new collagen I synthesis, markers of ECM metabolic turnover have not been evaluated. Future studies that incorporate these indicators are required to clarify this point. Fifth, we did not evaluate tissue inhibitors of metalloproteinases or MMP activity, limiting the interpretation of the detailed mechanisms regarding the effects of stretching and aging on ECM remodeling. Sixth, the aged mice represent a pre-atrophic stage, which may limit extrapolation to advanced sarcopenia. Finally, the sample size was smaller than that estimated by formal power analysis, which may have reduced the ability to detect small interaction effects. Therefore, nonsignificant findings should be interpreted with caution. Nevertheless, the consistent directionality of responses across time points and the presence of moderate to large effect sizes suggest that the observed changes reflect biologically meaningful adaptations rather than random variations. Despite these limitations, the present findings provide novel insights into the qualitative age-related alterations in ECM mechanosensitivity and remodeling capacity.

## 4. Materials and Methods

### 4.1. Animals

In this experiment, male C57BL/6J mice aged 12 weeks (young group, *n* = 16) and 72 weeks (aged group, *n* = 16) were used (Jackson Laboratory, Atsugi, Japan). No health issues were observed in the purchased animals. Prior to the experiment, the animals were acclimated for 1–2 weeks at the Hokuriku University Animal Research Facility. All animals had free access to food and water and were housed in standard transparent plastic cages. The animal facility maintained a controlled ambient temperature of 22 ± 2 °C and a 12-h light-dark cycle. The research protocol for this animal experiment was approved by the Hokuriku University Animal Experiment Committee (Approval No.: 24-14; Approval Date: 8 April 2024). All procedures complied with the Institutional Guidelines for the Use of Experimental Animals. In this experiment, we aimed to promptly alleviate any pain detected in animals during the procedure. Persistent recumbency or huddling, indicating impaired mobility, was defined as a humane endpoint and was used as an indicator for euthanasia. In addition, no animals were excluded from this experiment. This study was reported in accordance with the ARRIVE 2.0 guidelines.

### 4.2. Grouping

The young and aged groups were each divided into four groups. The STR0d group received no stretching (STR), whereas the STR3d, STR6d, and STR10d groups underwent stretching for 3, 6, and 10 days, respectively. The grouping was conducted randomly using a computer-generated random number sequence to assign animals to the control (STR0d) and stretching (STR3d, STR6d, and STR10d) groups within each age category. In this study, an a priori power analysis was performed using G*Power version 3.1 based on an assumed effect size of f = 0.4, an α error probability of 0.05, and a statistical power (1 − β) of 0.80, which indicated a required total sample size of 73 animals. However, because this study involved animal experimentation, the experimental design was carefully optimized in accordance with the principles of the 3Rs (replacement, reduction, and refinement). In addition, the availability of aged mice was limited. Considering these ethical and practical constraints, the study was conducted using a total of 32 mice. Four animals were assigned to each group. Accordingly, the present study was designed as an exploratory investigation to characterize age-related differences in ECM remodeling responses to repeated passive stretching. For hypothesis-testing purposes, the primary outcome measure used to determine the sample size was the collagen I IR area and intensity in the tibialis anterior muscle, which reflects stretch-induced intramuscular collagen remodeling.

### 4.3. Stretching

Stretching was performed on both the left and right tibialis anterior muscles of the mice. Specifically, mice were placed in a supine position on an animal heating pad under isoflurane anesthesia. The trunk was gently secured with white tape to stabilize posture during stretching without impairing respiration. Next, the mouse hindlimb crura were positioned in a dangling posture with the knee joint flexed, and the ankle joint plantar flexed. The knee joint, ankle joint, and base of the fifth metatarsal bone were vertically aligned in a straight line to apply stretching to both tibialis anterior muscles. A cycle consisting of 1 min of stretching followed by 30 s of rest was performed for 10 sets, in accordance with previous studies [15,16]. The joint angle-based stretching protocol was selected to enhance reproducibility and clinical relevance, as passive stretching in rehabilitation settings is commonly standardized by joint position rather than by direct force measurement. The tibialis anterior is a skeletal muscle with a predominance of fast-twitch fibers [29]. As sarcopenia primarily affects fast-twitch fibers [30], the tibialis anterior muscle was selected for this study. Furthermore, among the muscles of the hindlimb crura, the tibialis anterior possesses a moderate muscle mass, enabling multiple analyses. To minimize potential confounding factors, the order of stretching procedures and subsequent measurements was evenly distributed among the experimental groups. Blinding was not feasible during the intervention phase.

### 4.4. Sampling

Twenty-four hours after the final stretching session, the mice were weighed and euthanized by cervical dislocation. The tibialis anterior muscle was excised. The right tibialis anterior muscle was immediately immersed in RNAlater (Thermo Fisher Scientific, Waltham, MA, USA) and stored at 4 °C for 24 h. The left tibialis anterior muscle was weighed and rapidly frozen using isopentane and dry ice. The tibialis anterior muscles were stored at −80 °C until biochemical and histological analyses. To minimize potential confounding factors, tissue sampling was performed during the same time.

### 4.5. IF

From the central portion of the frozen tibialis anterior muscle, 10-µm-thick transverse sections were obtained using a cryostat (CM1950; Leica, Wetzlar, Germany) set at −25 °C and mounted on glass slides. Sections were fixed with 4% paraformaldehyde and washed with phosphate-buffered saline (PBS, pH 7.4). Sections were incubated at 4 °C for 1 h in PBS containing 1% normal goat serum and 0.3% Triton X-100. The sections were incubated overnight at 4 °C with rabbit polyclonal anti-collagen VI antibody (ab6588; Abcam, Cambridge, MA, USA) diluted 1:500 in PBS containing 0.3% Triton X-100. Next, the sections were incubated at 25 °C for 1 h with an Alexa Fluor 555-labeled anti-rabbit secondary antibody (4413S, Cell Signaling Technology, Danvers, MA, USA) diluted 1:1000 in PBS. After washing with PBS, the sections were embedded in a water-soluble embedding medium. Stained sections were observed under a microscope (BZ-X800; Keyence, Osaka, Japan).

### 4.6. IHC

From the central portion of the frozen tibialis anterior muscle, 10-µm-thick transverse sections were obtained using a cryostat (CM1950; Leica, Wetzlar, Germany) set at −25 °C and mounted on glass slides. Sections were fixed with 4% paraformaldehyde and washed with PBS (pH 7.4). Endogenous peroxidase activity was blocked by treating the sections with 3% hydrogen peroxide (H_2_O_2_), followed by washing with PBS and incubation at 4 °C for 1 h in PBS containing 1% normal goat serum and 0.3% Triton X-100. The sections were incubated overnight at 4 °C with rabbit polyclonal anti-collagen I, anti-collagen IV, anti-AGEs, or anti-LOX antibodies (ab21286, ab6586, ab23722, ab174316; Abcam, Cambridge, MA, USA) diluted 1:300–500 in PBS containing 0.3% Triton X-100. After primary antibody incubation, sections were reacted with biotinylated anti-rabbit immunoglobulin G (Vectastain ABC Kit; Vector Laboratories, Burlingame, CA, USA) diluted 1:1000 in PBS, at 25 °C for 1 h. Subsequently, the avidin–biotin complex (Vectastain ABC Kit) was incubated at 25 °C for 1 h. After washing with PBS, sections were rinsed with Tris-HCl buffer (pH 7.4) and incubated at 25 °C for 15 min with a 0.035% diaminobenzidine solution in Tris-HCl buffer containing 0.001% H_2_O_2_. After the DAB reaction, sections were counterstained with hematoxylin, dehydrated in a graded ethanol series, cleared with xylene, and mounted using Permount™ mounting medium (Pharma Co., Ltd., Tokyo, Japan).

### 4.7. Morphological Analysis

FCSA was measured using IF images with an anti-collagen VI antibody, following the BZ-X810 microscope manual (Keyence, Osaka, Japan), and analyzed using the Analysis Application Hybrid Cell Count from the same photograph. Over 280 muscle fibers were analyzed per sample. For IHC image analysis, semi-quantitative analysis was performed to evaluate IR intensity, IR area, and IR intensity/area using sections stained with anti-collagen I, anti-collagen IV, and anti-AGE antibodies. Image analysis was conducted using the Hybrid Cell Count analysis application of the BZ-X810 microscope system (Keyence, Osaka, Japan), in accordance with the manufacturer’s instructions. For each sample, three non-overlapping fields were analyzed, and the analyzed area per field was fixed at 393,880 μm^2^. IR intensity was defined as the integrated pixel intensity (sum of pixel values) within the positively stained area. IR area was expressed as the percentage of the positively stained area relative to the total analyzed area. IR intensity/area was calculated by dividing the integrated pixel intensity by the positively stained area. Changes in IR intensity and IR intensity/area were determined relative to those of the STR0d group of young mice. Furthermore, the number of LOX-positive muscle fibers and cells in the ECM was counted using three images per sample, each covering an area of 393,880 μm^2^. Cells positive for LOX in the ECM were identified based on LOX IR overlapping with the hematoxylin-stained nuclei. Muscle fibers with LOX IR in the cytoplasm were defined as LOX-positive muscle fibers. For images stained with each antibody, the photographs used for collagen I, AGE, and LOX analyses included the endomysium and perimysium regions, whereas those used for collagen IV analysis included the basement membrane region, consistent with primary localization. All image analyses were performed in a single-blind manner.

### 4.8. Quantitative PCR

Total RNA was extracted from the tissue samples using TRIzol reagent (Thermo Fisher Scientific, Waltham, MA, USA). The quality of the extracted RNA was assessed, and the RNA concentration was standardized to 1 μg for subsequent analysis. The first-strand cDNA was synthesized using random primers and ReverTra Ace (Toyobo, Osaka, Japan). Quantitative PCR was performed using TB Green Premix Ex Taq II (Takara Bio, Shiga, Japan) and the StepOnePlus Real-Time PCR System (Thermo Fisher Scientific, Waltham, MA, USA) under the following thermocycling conditions: an initial cycle of 30 s at 95 °C, followed by 40 cycles of 5 s at 95 °C and 30 s at 60 °C. A standard calibration curve was generated using cDNA templates, enabling the quantification of each target gene. Target gene expression levels were normalized to the expression of the housekeeping gene beta-2 microglobulin (*B2m*). The upregulation or downregulation of target genes was determined by comparing expression levels to those of the STR0d group of young mice. A detailed list of primers used in this study is shown in Table 1.

### 4.9. Western Blotting

MMP2 and MMP9 expression levels were evaluated using Western blotting. The frozen muscle tissue was homogenized in ice-cold homogenization buffer containing 50 mM Tris-HCl, 150 mM NaCl, 0.1% SDS, 1% NP-40, and 0.5% sodium deoxycholate (pH 8.0). A protease inhibitor cocktail (FUJIFILM Wako Pure Chemical Industries Ltd., Osaka, Japan) was added. The homogenate was centrifuged at 15,000 rpm for 5 min at 4 °C, and the supernatant was carefully collected for further analysis. Protein concentrations were measured using the DC Protein Assay Kit (Bio-Rad Laboratories, Hercules, CA, USA). The supernatant was solubilized in SDS sample buffer (10% glycerol, 2% SDS, 0.005% bromophenol blue, 100 mM dithiothreitol, and 50 mM Tris-HCl, pH 6.8) and heated at 98 °C for 3 min to ensure protein denaturation. Equal amounts of protein were separated by electrophoresis on 8% (for MMP2 and MMP9) or 10% (for glyceraldehyde-3-phosphate dehydrogenase; GAPDH) SDS–polyacrylamide gels and transferred to a polyvinylidene fluoride membrane. The membrane was incubated with 5% skim milk dissolved in Tris-buffered saline containing 0.1% Tween 20 to block nonspecific binding. The membrane was then incubated overnight at 4 °C with the primary antibody: rabbit monoclonal anti-MMP2 antibody (ab92536; Abcam, Cambridge, MA, USA) or rabbit polyclonal anti-MMP9 antibody (10375-2-AP; Proteintech Group, Inc., Rosemont, IL, USA). After incubation with the primary antibody, the membranes were further incubated with a horseradish peroxidase-labeled secondary antibody (Cell Signaling Technology, Beverly, MA, USA) at 25 °C for 1 h. GAPDH (ab181602; Abcam, Cambridge, MA, USA) was used as an internal loading control to confirm equal protein loading. Immunoreactive signals were visualized using a chemiluminescent detection system (Immunostar Zeta; Fujifilm Wako Pure Chemical Corporation, Osaka, Japan), and signal intensity was quantified using an image reader (FUSION Solo7; M&S Instruments Inc., Osaka, Japan). Digitized signal intensities were analyzed using ImageJ software (version 1.53, National Institutes of Health, Bethesda, MD, USA) [31].

### 4.10. Statistical Analysis

All statistical analyses were performed using SPSS version 28 (IBM SPSS Statistics, Japan; IBM, Tokyo, Japan). Two-way ANOVA was performed with age and stretching as factors. When a significant interaction was detected, Tukey’s post hoc test was applied for multiple comparisons. In the absence of a significant interaction, only main effects were interpreted. Statistical significance was set at *p* < 0.05. Results are presented as the mean with standard deviation. The statistical analyses were performed after confirming normality (Table S1) using the Shapiro–Wilk test with Holm correction via EZR (Saitama Medical Center, Saitama Medical University, Saitama, Japan) [32], a graphical user interface for R (version 4.2.1; R Foundation for Statistical Computing, Vienna, Austria).

## 5. Conclusions

We demonstrated that stretching induced an increase in collagen I IR area and intensity in both young and aged tibialis anterior muscles; however, age-related reduction of LOX-positive cells in the ECM suggests a diminished adaptive collagen cross-linking capacity. These findings suggest that, although the increase in fibrillar collagen in response to stretching is maintained with aging, the regulatory mechanisms controlling ECM stabilization are impaired. In addition, the aged mice represent a pre-atrophic stage, which may limit extrapolation to advanced sarcopenia. Therefore, the present findings may be most applicable to early aging stages and preventive strategies rather than to advanced sarcopenia.

## Figures and Tables

**Table 1:** Primer sequences utilized in this study.

Genes	Direction	Nucleotide Positions and Sequence (5′-3′)	Sequence ID
*Col1a1*	Forward	2916 GATCTCCTGGTGCTGATG 2933	NM_007742.4
	Reverse	3028 GAAGCCTCTTTCTCCTCTCTGA 3007	NM_007742.4
*Col4a1*	Forward	4819 TGTCCATGGCACCCATCTC 4837	NM_009931.2
	Reverse	4912 CTGTGTACCGCCATCACCA 4894	NM_009931.2
*Lox*	Forward	890 GTGCCCGACCCCTACTACAT 909	M65142.1
	Reverse	1007 TGACATCCGCCCTATATGCT 988	M65142.1
*Mmp2*	Forward	2120 AAGAAAATGGACCCCGGTTT 2139	NM_008610.3
	Reverse	2251 CTTCAGGTAATAAGCACCCTTG 2230	NM_008610.3
*Mmp9*	Forward	885 TGTTCCCGTTCATCTTTGAG 904	NM_013599.5
	Reverse	988 ATCCTGGTCATAGTTGGCTGT 968	NM_013599.5
*B2m*	Forward	77 TTCTGGTGCTTGTCTCACTGA 97	NM_009735.3
	Reverse	180 CAGTATGTTCGGCTTCCCATTC 159	NM_009735.3

## Data Availability

The data presented in this study are available in Figshare at 10.6084/m9.figshare.31272367 (posted on 15 March 2026).

## Supplementary Materials

The following supporting information can be downloaded at: https://www.mdpi.com/article/10.3390/ijms27062753/s1.

## Abbreviations

The following abbreviations are used in this manuscript:

ECM	Extracellular matrix
LOX	Lysyl oxidase
AGEs	Advanced glycation end products
MMPs	Matrix metalloproteinases
FCSA	Fiber cross-sectional area
IF	Immunofluorescence
IHC	Immunohistochemistry
IR	Immunoreactivity
B2m	Beta-2 microglobulin
GAPDH	Glyceraldehyde-3-phosphate dehydrogenase
