# Supplementary material for: Aging Impairs Intramuscular Collagen Remodeling Responses to Repeated Passive Stretching in Skeletal Muscle
Source: Int J Mol Sci. 2026 Mar 18;27(6):2753. doi: 10.3390/ijms27062753 (PMC13027270; doi:10.3390/ijms27062753)
Supplement: Supplementary file 1 [file ijms-27-02753-s001.zip › Supplemental Figures.pdf]

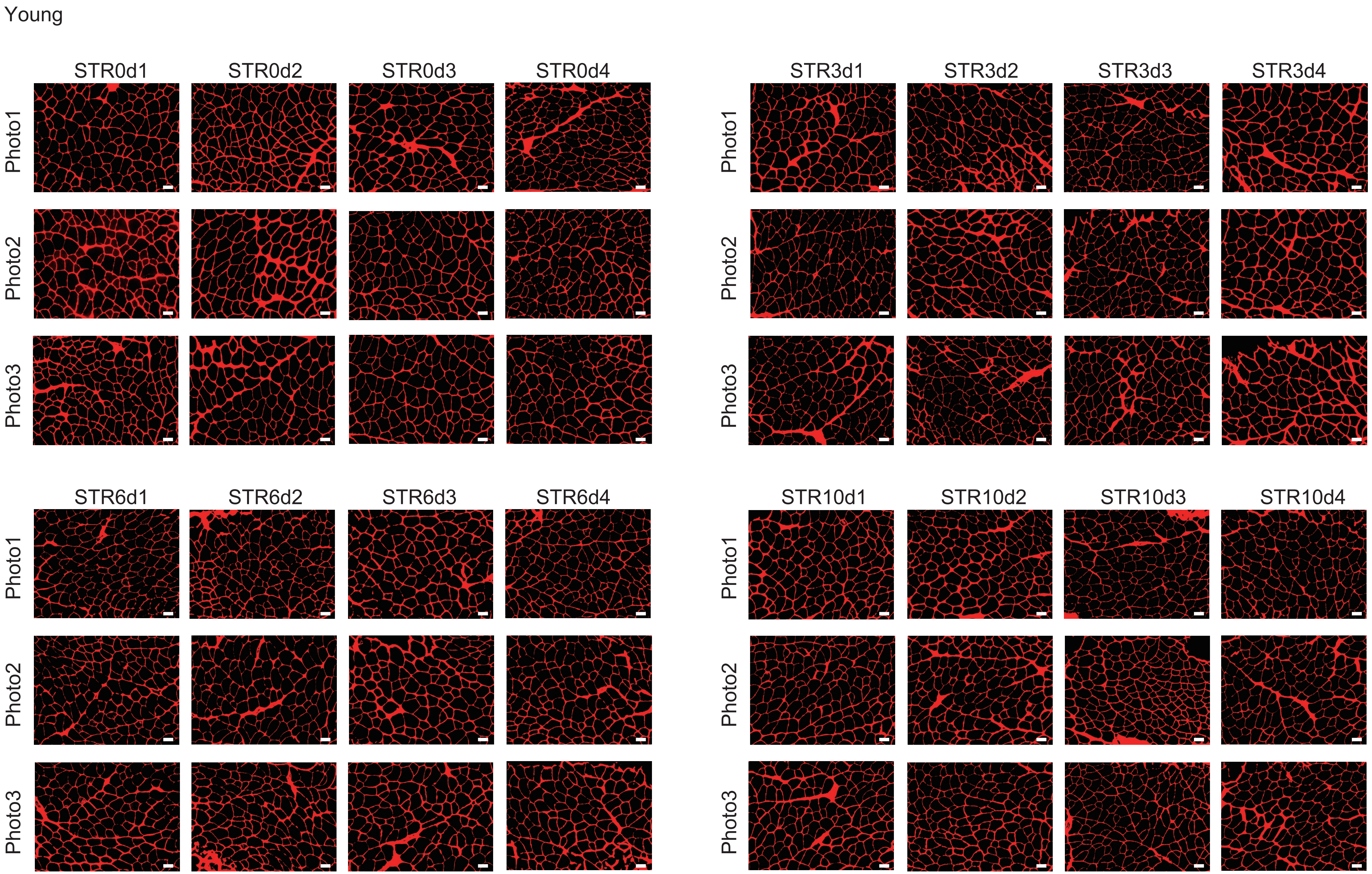

Supplementary Figure S1 Fiber cross-sectional area in young mice. Transverse sections of the tibialis anterior muscle were prepared and visualized using immunofluorescence with anti-collagen VI antibody to outline muscle fibers. The scale bar indicates 50 μm. Fiber cross-sectional area was measured using the stained images. STR0d: No stretching, STR3d: Stretching for 3 days, STR6d: Stretching for 6 days, STR10d: Stretching for 10 days.

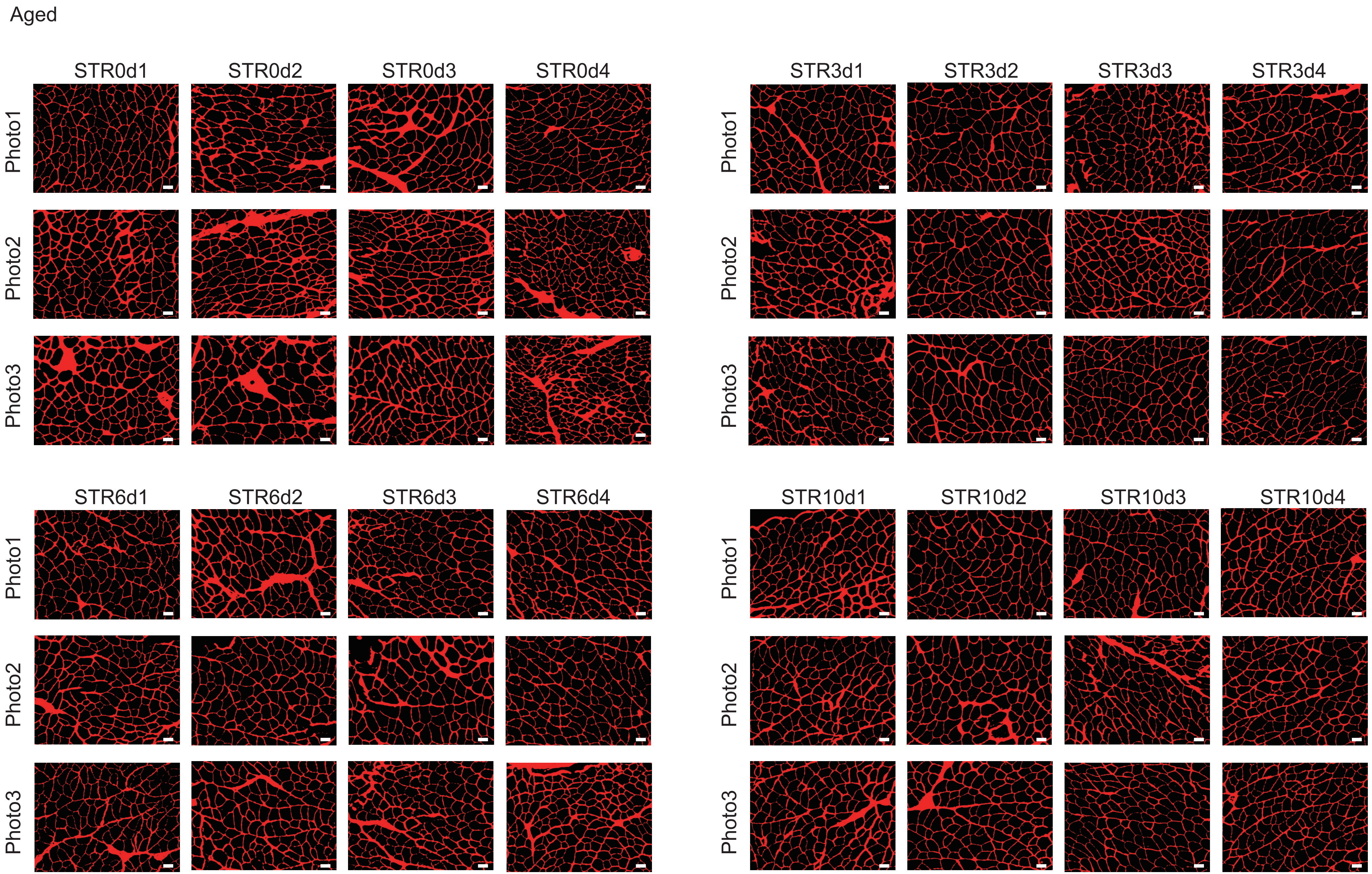

Supplementary Figure S2 Fiber cross-sectional area in aged mice. Transverse sections of the tibialis anterior muscle were prepared and visualized using immunofluorescence with anti-collagen VI antibody to outline muscle fibers. The scale bar indicates 50 μm. Fiber cross-sectional area was measured using the stained images. STR0d: No stretching, STR3d: Stretching for 3 days, STR6d: Stretching for 6 days, STR10d: Stretching for 10 days.

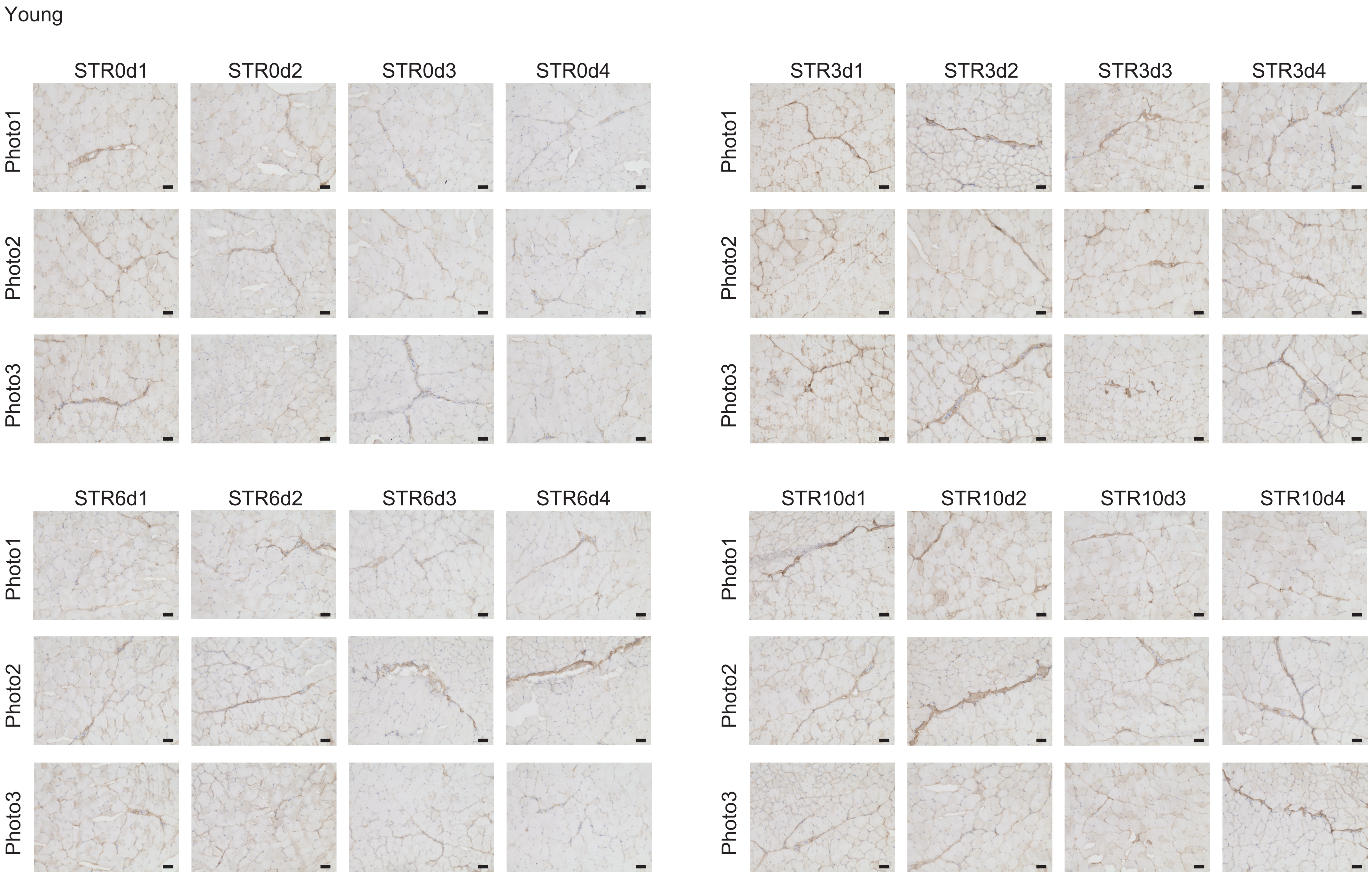

Supplementary Figure S3 Collagen I localization in young mice. Collagen I was visualized by immunohistochemical staining with anti-collagen I antibody in cross-sections of the tibialis anterior muscle, with hematoxylin used for counterstaining. Scale bar = 50 μm. STR0d: No stretching, STR3d: Stretching for 3 days, STR6d: Stretching for 6 days, STR10d: Stretching for 10 days.

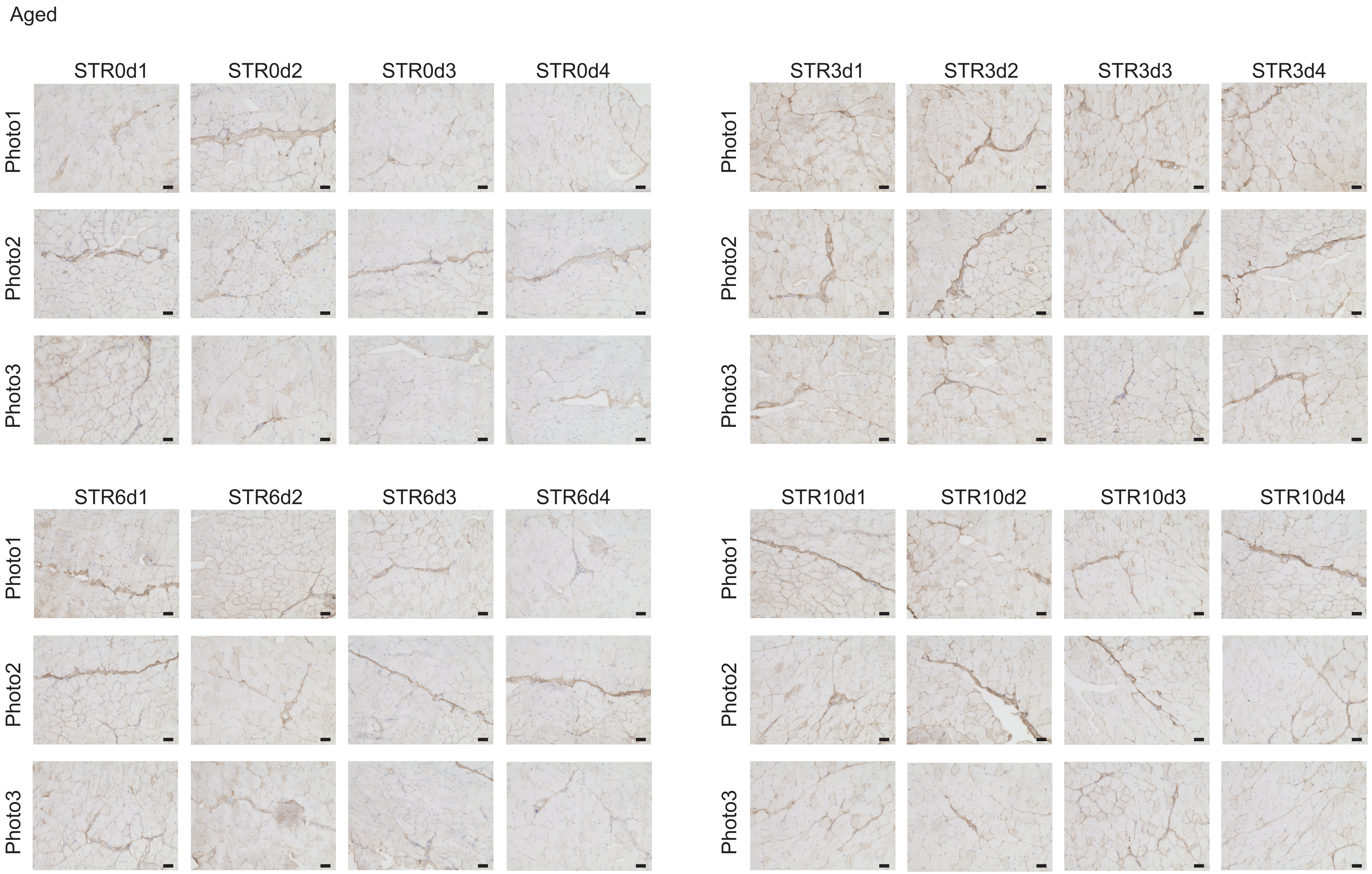

Supplementary Figure S4 Collagen I localization in aged mice. Collagen I was visualized by immunohistochemical staining with anti-collagen I antibody in cross-sections of the tibialis anterior muscle, with hematoxylin used for counterstaining. Scale bar = 50 μm. STR0d: No stretching, STR3d: Stretching for 3 days, STR6d: Stretching for 6 days, STR10d: Stretching for 10 days.

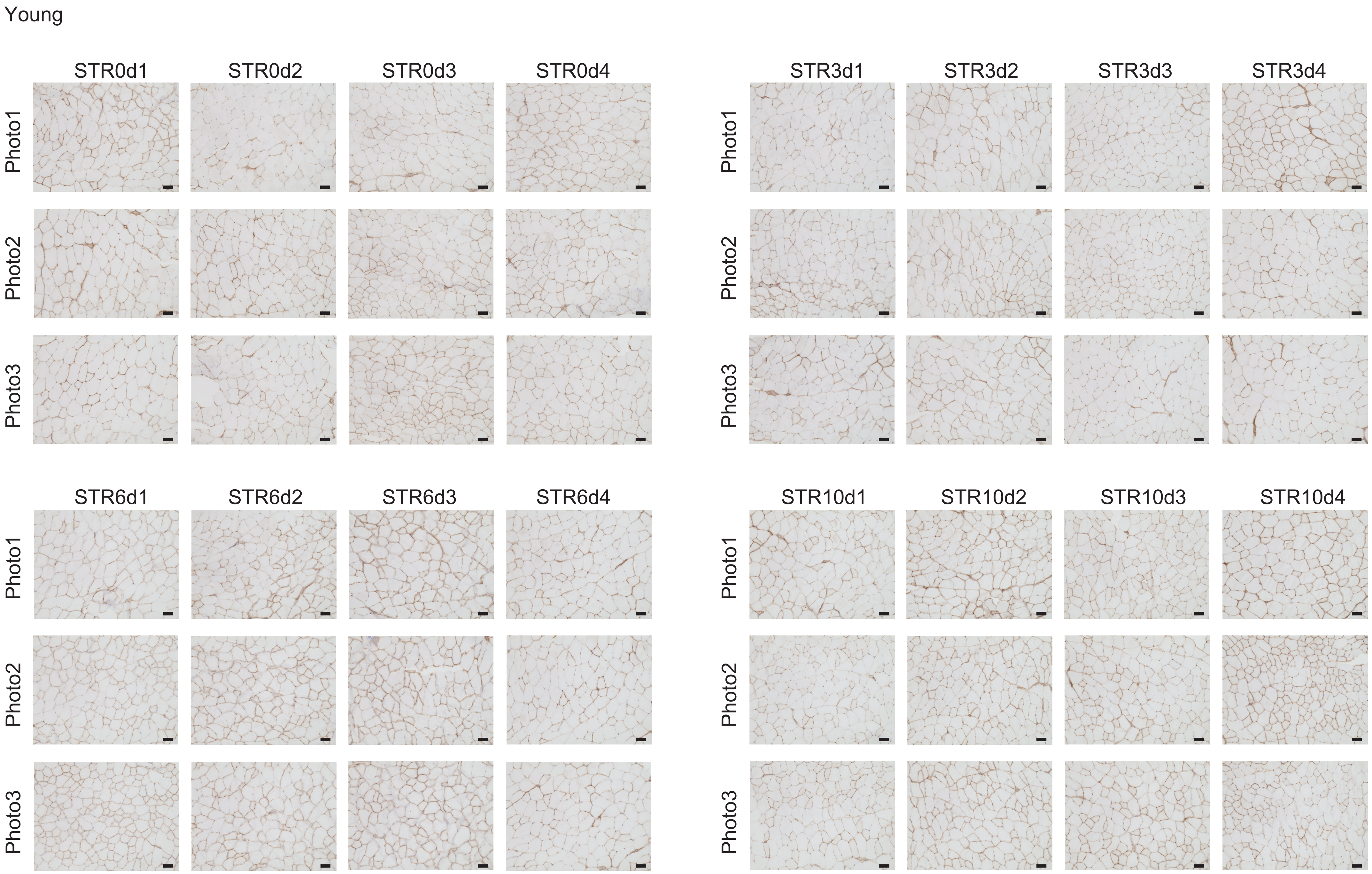

Supplementary Figure S5 Collagen IV localization in young mice. Collagen IV was visualized by immunohistochemical staining with anti-collagen IV antibody in cross-sections of the tibialis anterior muscle, with hematoxylin used for counterstaining. Scale bar = 50 μm. STR0d: No stretching, STR3d: Stretching for 3 days, STR6d: Stretching for 6 days, STR10d: Stretching for 10 days.

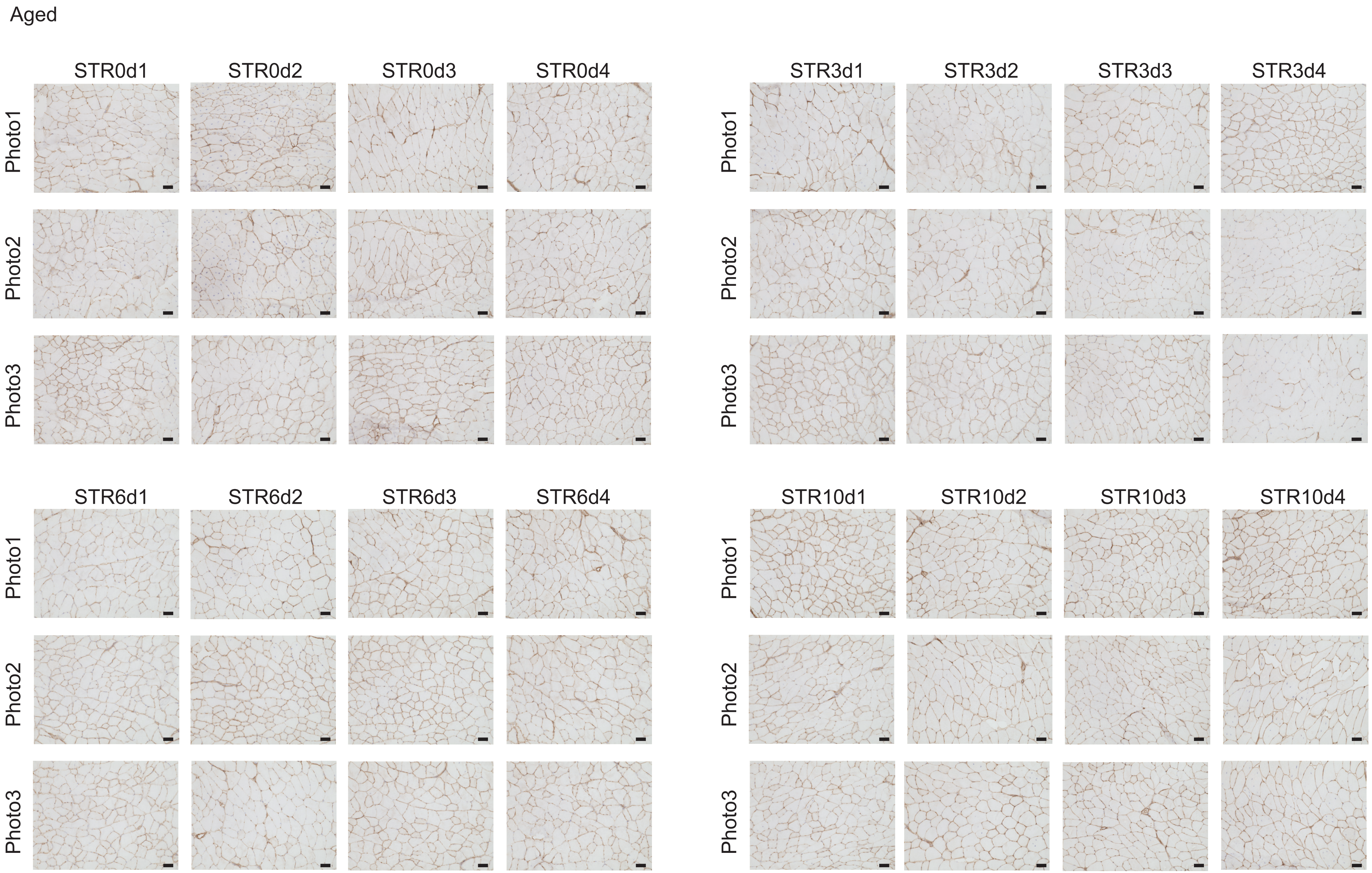

Supplementary Figure S6 Collagen IV localization in aged mice. Collagen IV was visualized by immunohistochemical staining with anti-collagen IV antibody in cross-sections of the tibialis anterior muscle, with hematoxylin used for counterstaining. Scale bar = 50 μm. STR0d: No stretching, STR3d: Stretching for 3 days, STR6d: Stretching for 6 days, STR10d: Stretching for 10 days.

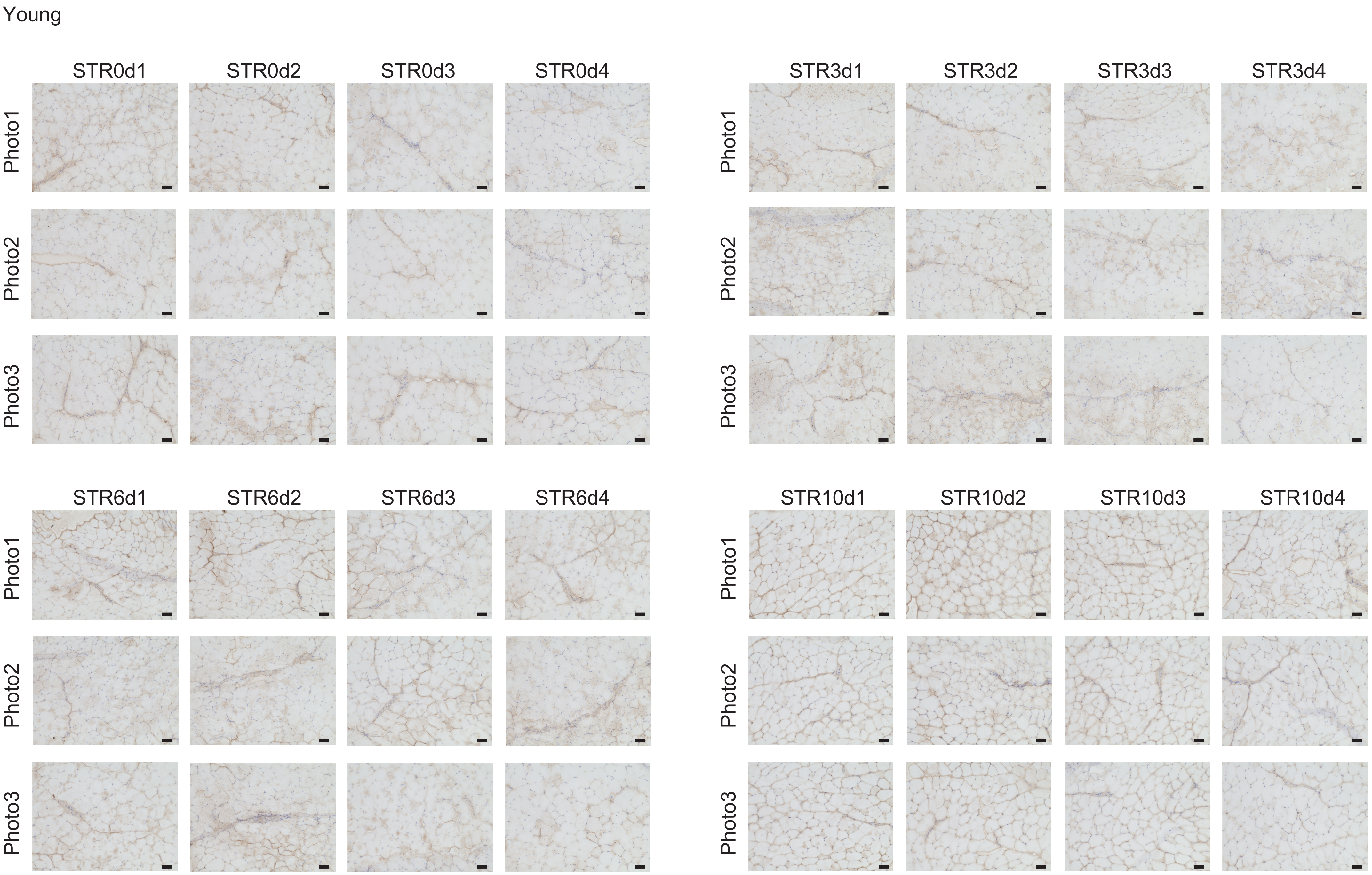

Supplementary Figure S7 AGE localization in young mice. AGEs were visualized by immunohistochemical staining with anti-AGE antibody in cross-sections of the tibialis anterior muscle, with hematoxylin used for counterstaining. Scale bar = 50 μm. STR0d: No stretching, STR3d: Stretching for 3 days, STR6d: Stretching for 6 days, STR10d: Stretching for 10 days.

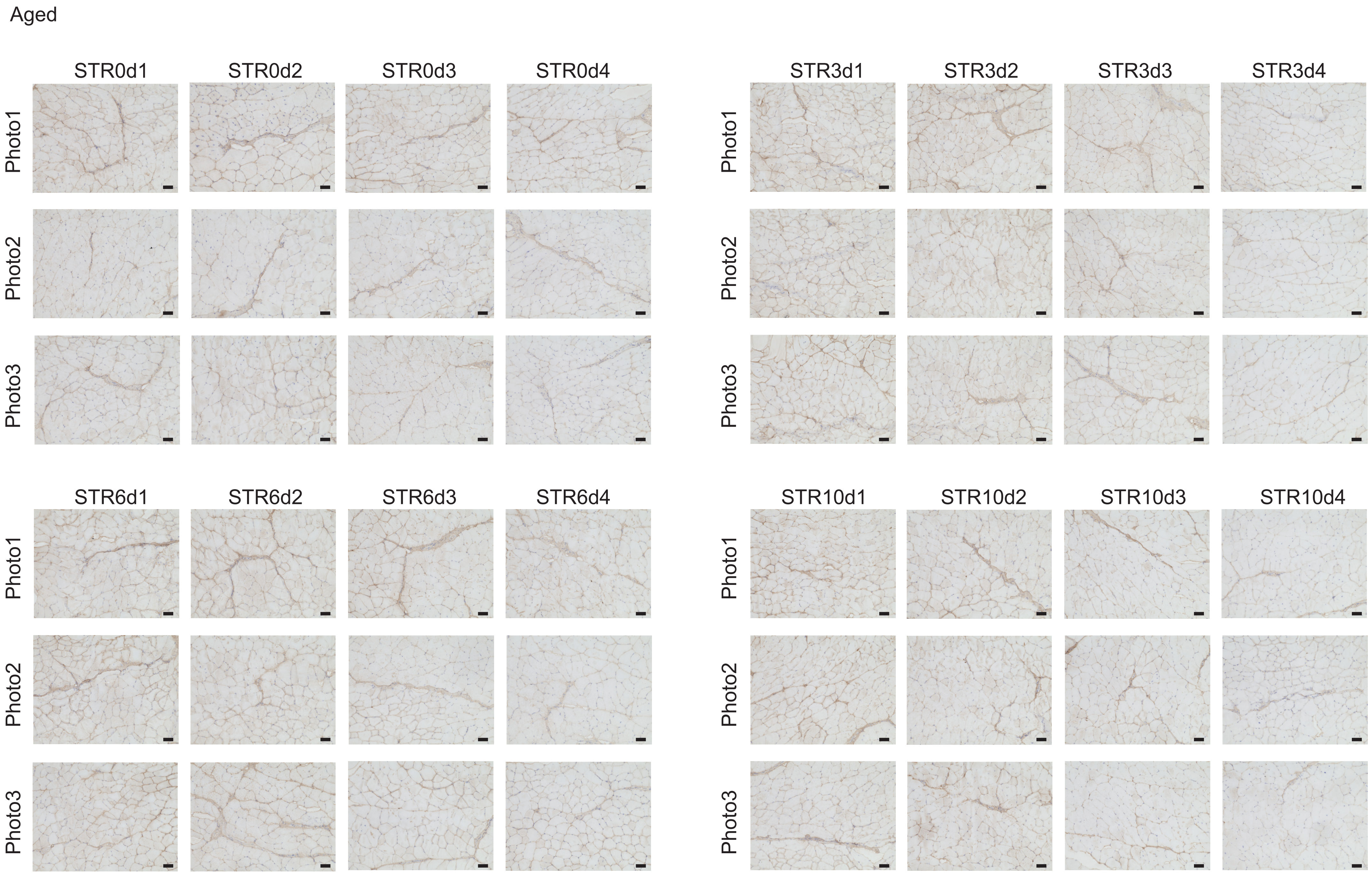

Supplementary Figure S8 AGE localization in aged mice. AGEs were visualized by immunohistochemical staining with anti-AGE antibody in cross-sections of the tibialis anterior muscle, with hematoxylin used for counterstaining. Scale bar = 50 μm. STR0d: No stretching, STR3d: Stretching for 3 days, STR6d: Stretching for 6 days, STR10d: Stretching for 10 days.

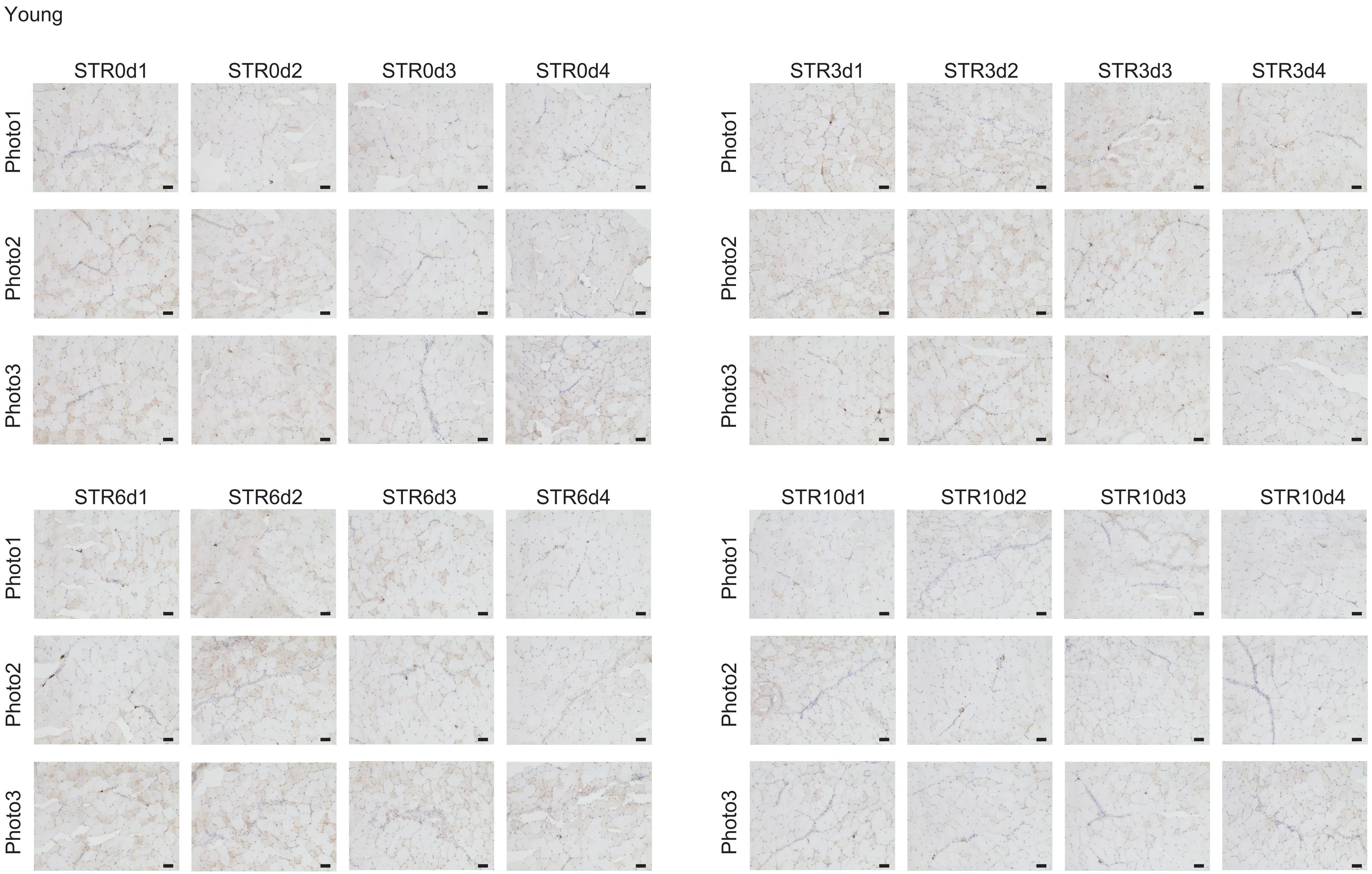

Supplementary Figure S9 LOX localization in young mice. LOX were visualized by immunohistochemical staining with anti-LOX antibody in cross-sections of the tibialis anterior muscle, with hematoxylin used for counterstaining. Scale bar = 50 μm. STR0d: No stretching, STR3d: Stretching for 3 days, STR6d: Stretching for 6 days, STR10d: Stretching for 10 days.

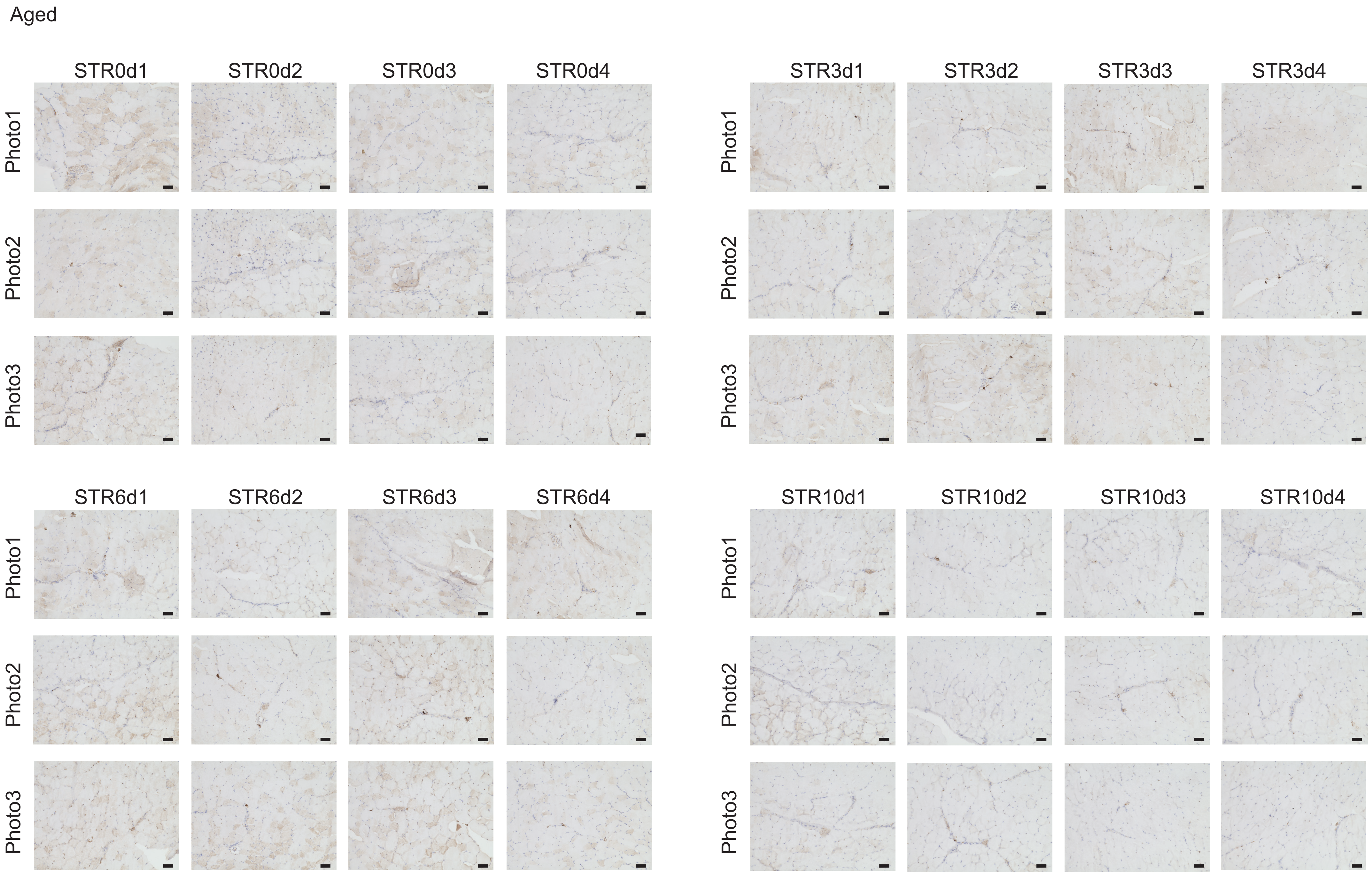

Supplementary Figure S10 LOX localization in aged mice. LOX were visualized by immunohistochemical staining with anti-LOX antibody in cross-sections of the tibialis anterior muscle, with hematoxylin used for counterstaining. Scale bar = 50 µm. STR0d: No stretching, STR3d: Stretching for 3 days, STR6d: Stretching for 6 days, STR10d: Stretching for 10 days.

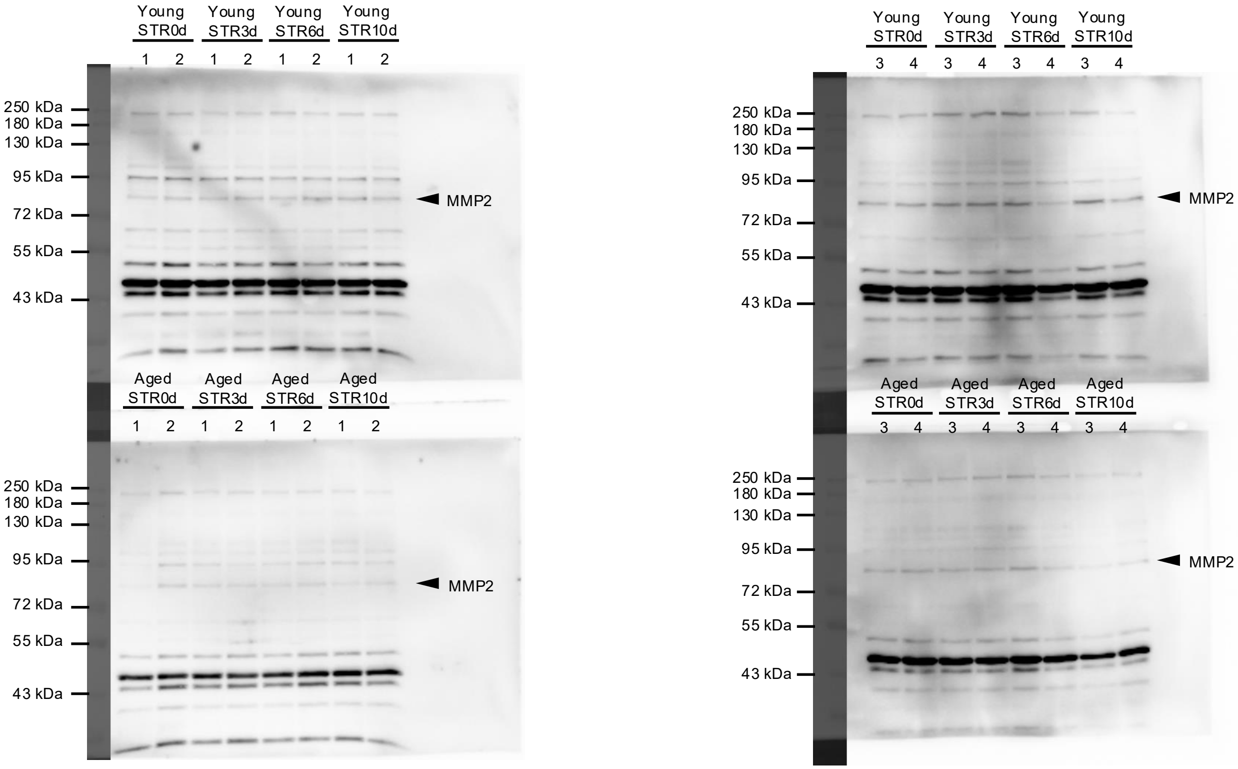

Supplementary Figure S11 MMP2 expression. Using the tibialis anterior muscle, MMP2 expression levels were measured by western blotting. STR0d: No stretching, STR3d: Stretching for 3 days, STR6d: Stretching for 6 days, STR10d: Stretching for 10 days.

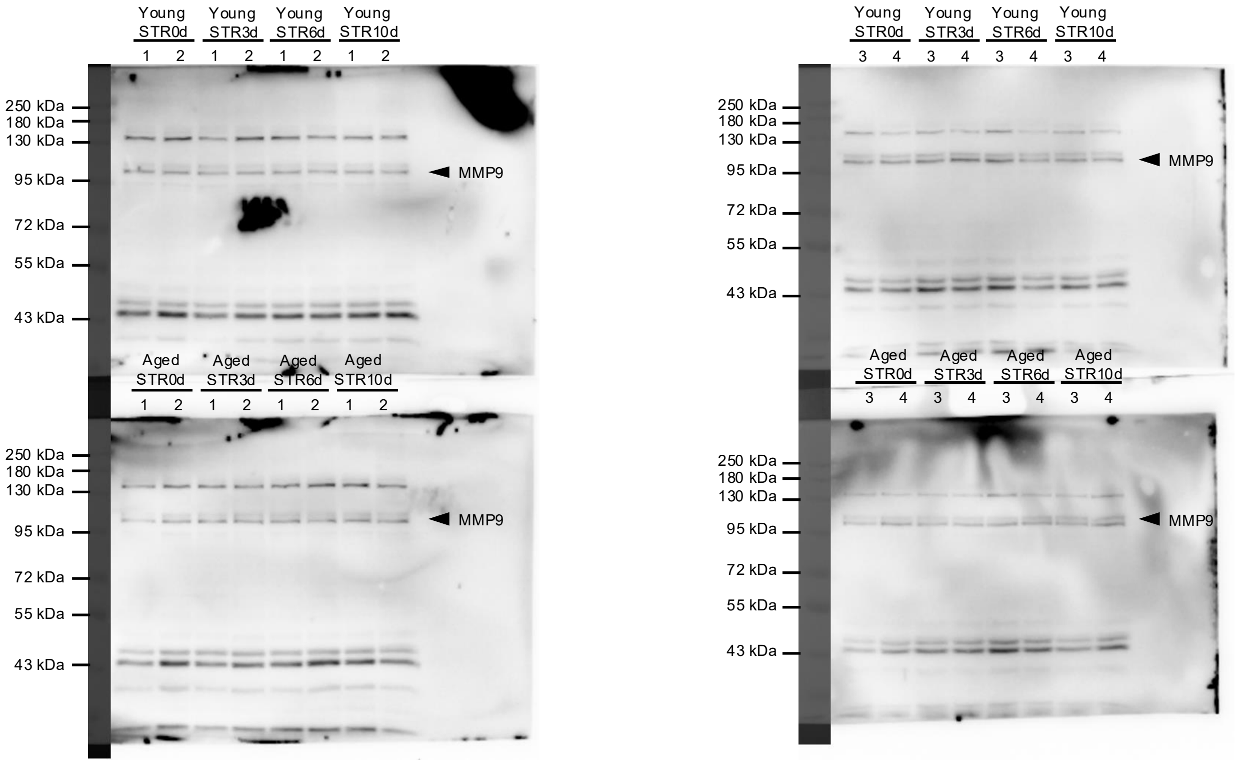

Supplementary Figure S12 MMP9 expression. Using the tibialis anterior muscle, MMP9 expression levels were measured by western blotting. STR0d: No stretching, STR3d: Stretching for 3 days, STR6d: Stretching for 6 days, STR10d: Stretching for 10 days.

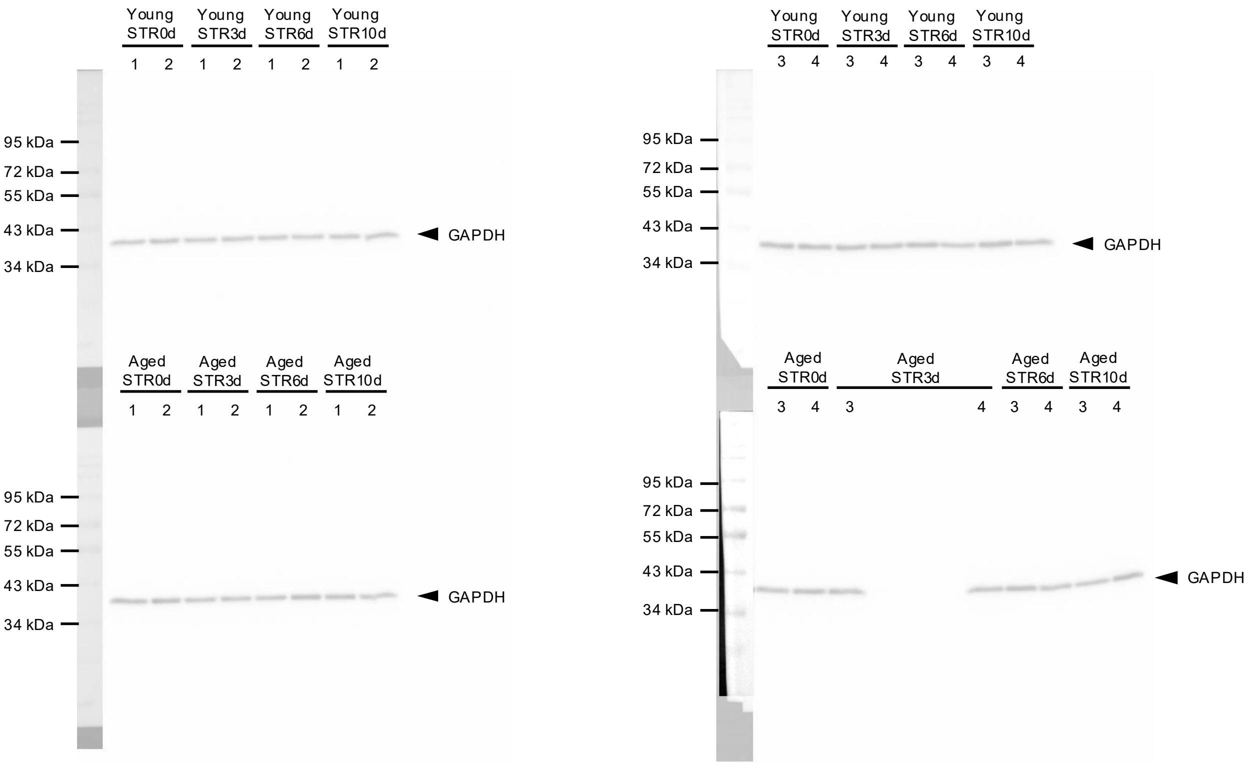

Supplementary Figure S13 GAPDH expression. Using the tibialis anterior muscle, GAPDH expression levels were measured by western blotting. STR0d: No stretching, STR3d: Stretching for 3 days, STR6d: Stretching for 6 days, STR10d: Stretching for 10 days.

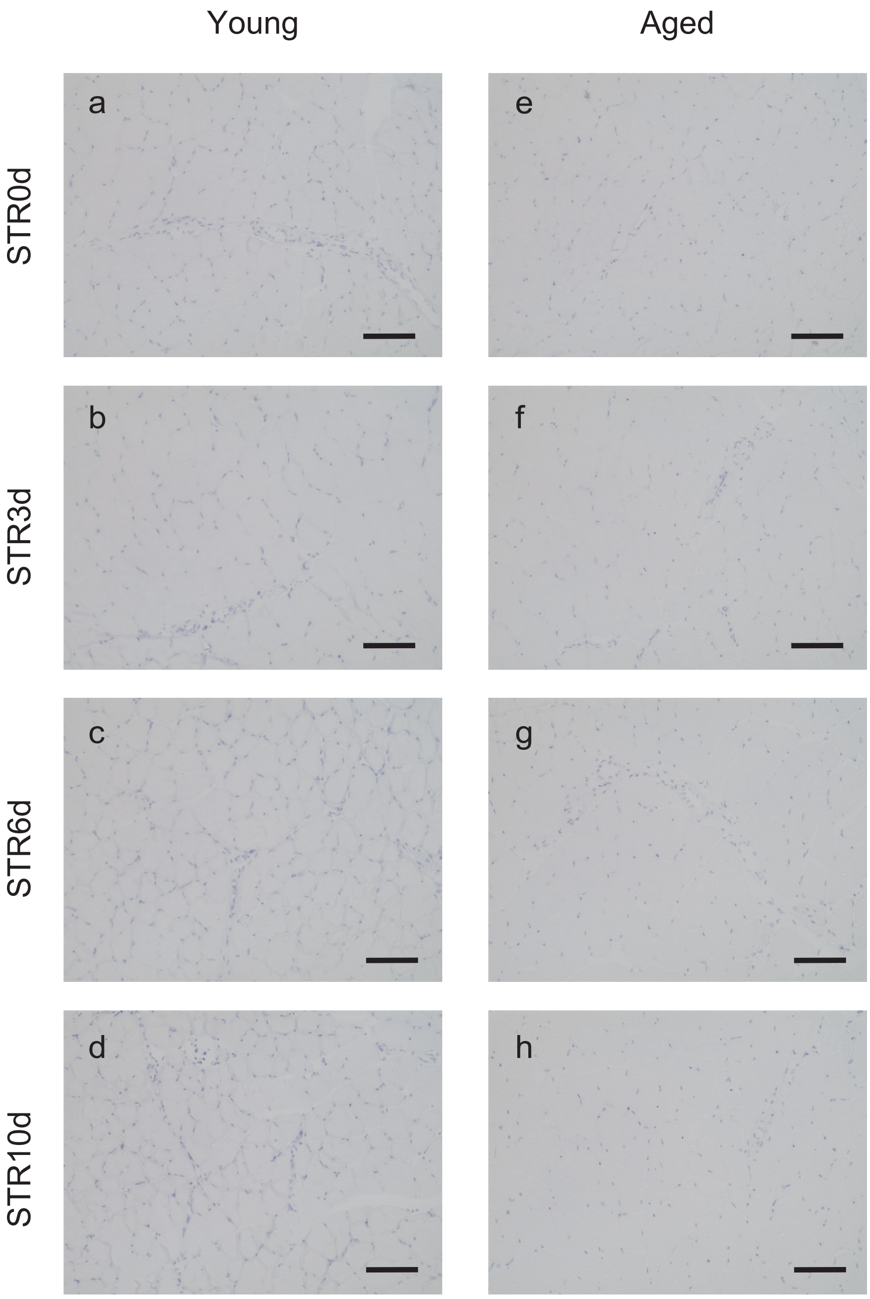

Supplementary Figure S14 Negative control staining of tibialis anterior muscle sections. Representative images of transverse sections of tibialis anterior muscle from Young (a–d) and Aged (e–h) groups at STR0d (a, e), STR3d (b, f), STR6d (c, g), and STR10d (d, h). Sections were processed identically to the immunohistochemical protocol but incubated without primary antibodies. No detectable DAB-positive signal was observed in any group. Sections were counterstained with hematoxylin. The scale bar indicates 100 μm. STR0d: No stretching, STR3d: Stretching for 3 days, STR6d: Stretching for 6 days, STR10d: Stretching for 10 days.

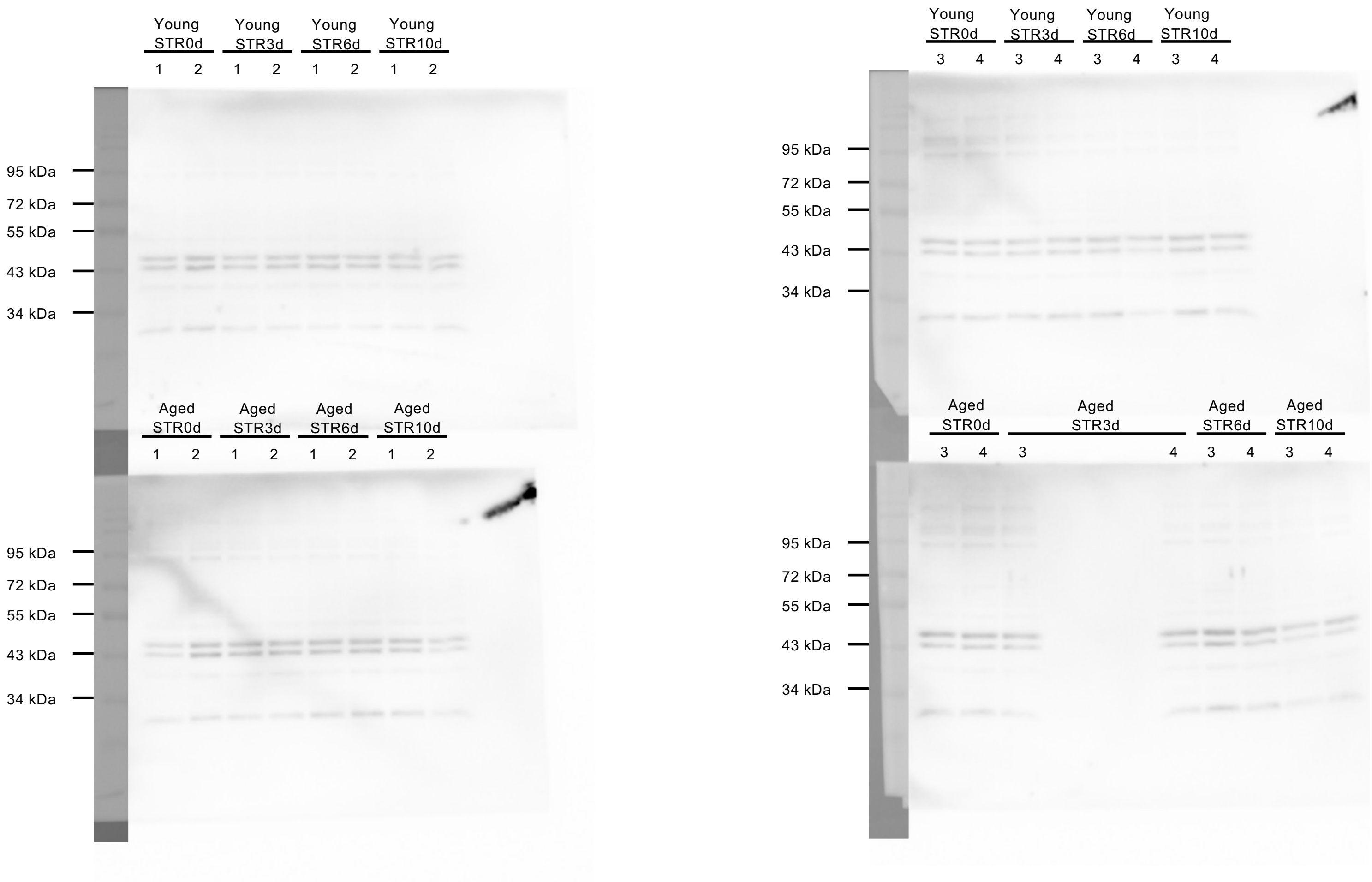

Supplementary Figure S15 Negative control for Western blot. Membranes were incubated with rabbit IgG instead of the primary antibody, followed by HRP-conjugated anti-rabbit IgG secondary antibody. Non-specific background bands were observed. STR0d: No stretching, STR3d: Stretching for 3 days, STR6d: Stretching for 6 days, STR10d: Stretching for 10 days.
