# Supplementary material for: Aging Impairs Intramuscular Collagen Remodeling Responses to Repeated Passive Stretching in Skeletal Muscle
Source: Int J Mol Sci. 2026 Mar 18;27(6):2753. doi: 10.3390/ijms27062753 (PMC13027270; doi:10.3390/ijms27062753)
Supplement: Supplementary file 1 [file ijms-27-02753-s001.zip › Table S1.pdf]

Table S1. Confirmation of normality

Group	Experimental data	Adjusted p-value
Young + STR10d	*Mmp9*	0.996
Young + STR10d	AGEs-IR area	1.966
Young + STR10d	*Col4a1*	2.940
Aged + STR10d	LOX (+) cells in ECM	3.892
Young + STR10d	*Lox*	4.835
Aged + STR3d	AGEs-IR intensity	5.799
Aged + STR6d	*Mmp9*	6.710
Young + STR10d	MMP2 expression (WB)	7.533
Aged + STR10d	Muscle weight	8.469
Aged + STR10d	MMP9 expression (WB)	9.364
Aged + STR0d	*Mmp2*	10.098
Young + STR3d	*Col4a1*	10.944
Young + STR0d	Collagen Ⅳ-IR intensity/area	11.804
Young + STR0d	Muscle weight	12.600
Aged + STR6d	AGEs-IR intensity	13.457
Aged + STR6d	Collagen I-IR intensity	14.232
Aged + STR6d	AGEs-IR area	15.096
Young + STR6d	Muscle weight	15.678
Young + STR3d	AGEs-IR area	16.530
Aged + STR3d	AGEs-IR area	17.220
Young + STR0d	LOX (+) cells in ECM	18.081
Aged + STR10d	Collagen I-IR area	18.920
Aged + STR6d	*Col4a1*	19.412
Aged + STR10d	Collagen I-IR intensity	20.218
Aged + STR6d	Collagen I-IR area	21.000
Young + STR10d	AGEs-IR intensity	21.765
Aged + STR10d	*Mmp2*	22.572
Young + STR6d	Collagen Ⅳ-IR intensity	23.372
Young + STR10d	Relative muscle weight	24.128
Young + STR3d	MMP2 expression (WB)	24.897
Aged + STR10d	Body weight	25.296

Aged + STR3d	LOX (+) muscle fiber	25.542
Aged + STR10d	AGEs-IR area	25.938
Aged + STR0d	MMP9 expression (WB)	26.619
Young + STR0d	FCSA	27.370
Aged + STR3d	Collagen I-IR area	27.792
Young + STR6d	Collagen Ⅳ-IR area	28.416
Young + STR6d	Collagen I-IR intensity	29.089
Aged + STR3d	AGEs-IR intensity/area	29.710
Aged + STR0d	Body weight	30.360
Young + STR10d	Collagen I-IR intensity	30.541
Aged + STR6d	*Mmp2*	31.206
Aged + STR6d	Collagen Ⅳ-IR intensity	31.850
Young + STR10d	Collagen I-IR area	32.560
Aged + STR3d	Collagen I-IR intensity	33.228
Young + STR3d	Collagen I-IR intensity/area	33.847
Young + STR3d	MMP9 expression (WB)	34.146
Young + STR6d	AGEs-IR intensity	34.253
Aged + STR6d	Collagen I-IR intensity/area	34.952
Aged + STR0d	AGEs-IR area	35.600
Aged + STR0d	Collagen Ⅳ-IR intensity/area	35.930
Aged + STR6d	Collagen Ⅳ-IR area	36.348
Young + STR10d	LOX (+) muscle fiber	36.199
Aged + STR6d	AGEs-IR intensity/area	36.801
Aged + STR6d	LOX (+) cells in ECM	37.455
Young + STR10d	MMP9 expression (WB)	38.024
Young + STR10d	Collagen Ⅳ-IR area	38.646
Aged + STR6d	MMP2 expression (WB)	39.173
Aged + STR0d	*Col1a1*	39.589
Aged + STR10d	AGEs-IR intensity	40.128
Young + STR3d	Collagen Ⅳ-IR intensity/area	40.358
Young + STR6d	Collagen I-IR intensity/area	40.852
Young + STR6d	Collagen I-IR area	40.824
Aged + STR3d	*Col4a1*	40.832
Aged + STR3d	Muscle weight	41.470

Young + STR3d	Collagen I-IR area	41.844
Young + STR6d	AGEs-IR area	41.942
Young + STR0d	*Mmp9*	42.072
Young + STR6d	Body weight	42.504
Young + STR0d	*Col1a1*	42.350
Young + STR0d	MMP2 expression (WB)	42.884
Young + STR3d	Collagen I-IR intensity	42.898
Young + STR10d	Collagen Ⅳ-IR intensity	43.311
Young + STR3d	*Mmp2*	43.808
Aged + STR3d	*Col1a1*	43.725
Young + STR10d	LOX (+) cells in ECM	43.472
Aged + STR0d	*Mmp9*	43.243
Young + STR6d	*Col4a1*	43.758
Aged + STR0d	FCSA	44.240
Aged + STR3d	LOX (+) cells in ECM	44.320
Young + STR0d	MMP9 expression (WB)	44.866
Aged + STR10d	Collagen Ⅳ-IR intensity	45.346
Aged + STR10d	Collagen Ⅳ-IR area	44.820
Young + STR3d	*Mmp9*	43.680
Aged + STR10d	FCSA	43.265
Young + STR6d	LOX (+) cells in ECM	43.688
Aged + STR6d	MMP9 expression (WB)	44.031
Young + STR10d	Body weight	44.528
Young + STR3d	*Lox*	44.856
Young + STR6d	*Mmp9*	45.180
Aged + STR0d	AGEs-IR intensity	43.598
Aged + STR10d	*Mmp9*	43.479
Aged + STR10d	MMP2 expression (WB)	43.691
Aged + STR3d	*Mmp2*	43.710
Aged + STR0d	MMP2 expression (WB)	43.881
Aged + STR0d	Collagen I-IR area	44.064
Young + STR3d	FCSA	43.941
Aged + STR6d	FCSA	44.296
Aged + STR0d	Collagen I-IR intensity	44.184

Aged + STR0d	*Lox*	44.100
Young + STR10d	Muscle weight	43.834
Young + STR6d	Relative muscle weight	43.248
Young + STR0d	Collagen I-IR intensity/area	43.456
Aged + STR0d	Collagen Ⅳ-IR area	43.264
Young + STR0d	Collagen Ⅳ-IR area	43.260
Young + STR0d	*Mmp2*	42.824
Young + STR3d	Relative muscle weight	42.479
Young + STR10d	Collagen I-IR intensity/area	42.725
Young + STR10d	Collagen Ⅳ-IR intensity/area	41.987
Aged + STR3d	Relative muscle weight	42.020
Young + STR3d	AGEs-IR intensity	42.391
Aged + STR3d	MMP2 expression (WB)	42.302
Aged + STR3d	*Lox*	42.149
Aged + STR6d	LOX (+) muscle fiber	42.066
Aged + STR3d	Collagen I-IR intensity/area	42.424
Aged + STR0d	Collagen Ⅳ-IR intensity	42.572
Aged + STR3d	FCSA	42.939
Young + STR0d	AGEs-IR intensity/area	42.338
Young + STR0d	Body weight	42.364
Aged + STR3d	MMP9 expression (WB)	42.180
Aged + STR3d	*Mmp9*	40.959
Aged + STR6d	Body weight	41.114
Young + STR3d	Collagen Ⅳ-IR area	40.713
Aged + STR10d	*Col4a1*	40.424
Young + STR3d	*Col1a1*	40.000
Young + STR6d	*Mmp2*	37.800
Aged + STR10d	Collagen Ⅳ-IR intensity/area	37.973
Young + STR0d	*Col4a1*	36.992
Young + STR0d	*Lox*	36.894
Young + STR0d	Relative muscle weight	36.530
Young + STR6d	MMP9 expression (WB)	36.654
Aged + STR10d	AGEs-IR intensity/area	36.604
Aged + STR6d	*Col1a1*	36.841

Young + STR6d	Collagen Ⅳ-IR intensity/area	36.997
Aged + STR0d	AGEs-IR intensity/area	37.139
Young + STR6d	LOX (+) muscle fiber	37.060
Young + STR3d	LOX (+) muscle fiber	37.127
Young + STR0d	Collagen Ⅳ-IR intensity	37.094
Aged + STR10d	Relative muscle weight	37.252
Young + STR10d	*Mmp2*	36.400
Young + STR6d	FCSA	34.263
Aged + STR3d	Body weight	34.222
Aged + STR10d	*Lox*	34.177
Aged + STR0d	Muscle weight	34.128
Aged + STR6d	Muscle weight	34.365
Aged + STR10d	LOX (+) muscle fiber	32.733
Aged + STR6d	Relative muscle weight	32.634
Aged + STR6d	Collagen Ⅳ-IR intensity/area	32.545
Aged + STR10d	Collagen I-IR intensity/area	29.994
Aged + STR0d	LOX (+) muscle fiber	29.175
Young + STR6d	AGEs-IR intensity/area	28.932
Young + STR0d	Collagen I-IR intensity	29.078
Aged + STR0d	LOX (+) cells in ECM	28.917
Young + STR0d	Collagen I-IR area	28.490
Young + STR0d	AGEs-IR area	27.280
Young + STR3d	Collagen Ⅳ-IR intensity	26.988
Young + STR0d	AGEs-IR intensity	26.926
Aged + STR0d	Collagen I-IR intensity/area	20.366
Young + STR6d	MMP2 expression (WB)	17.729
Young + STR3d	Body weight	17.440
Aged + STR0d	*Col4a1*	14.168
Young + STR3d	Muscle weight	11.664
Aged + STR3d	Collagen Ⅳ-IR intensity	11.694
Aged + STR3d	Collagen Ⅳ-IR area	10.824
Young + STR3d	AGEs-IR intensity/area	9.888
Young + STR6d	*Lox*	9.794
Aged + STR10d	*Col1a1*	9.185

Young + STR10d	*Col1a1*	9.240
Young + STR10d	FCSA	8.619
Aged + STR6d	*Lox*	8.330
Young + STR3d	LOX (+) cells in ECM	4.446
Aged + STR0d	Relative muscle weight	3.612
Young + STR6d	*Col1a1*	2.249
Young + STR10d	AGEs-IR intensity/area	2.133
Aged + STR3d	Collagen Ⅳ-IR intensity/area	0.756
Young + STR0d	LOX (+) muscle fiber	0.218

The *p*-values by the Shapiro-Wilk test were presented as adjusted *p*-values by the Holm method.
